# Brightening New Horizons: Luminescent Transition Metal Complexes in Optical Imaging and Theranostic Applications

**DOI:** 10.1021/acscentsci.5c00975

**Published:** 2025-07-16

**Authors:** Lawrence Cho-Cheung Lee, Kenneth Kam-Wing Lo

**Affiliations:** † Department of Chemistry, 53025 City University of Hong Kong, Tat Chee Avenue, Kowloon, Hong Kong, P. R. China; ‡ State Key Laboratory of Terahertz and Millimeter Waves, 53025 City University of Hong Kong, Tat Chee Avenue, Kowloon, Hong Kong, P. R. China

## Abstract

Small-molecule fluorescent probes have revolutionized fluorescence imaging in both biological research and clinical applications, allowing the exploration of intricate cellular processes and aiding disease diagnosis and treatment. Advances in the designs of molecular probes have led to the development of fluorescent theranostics that combine therapeutic and diagnostic modalities in a single platform. These dual-functional agents enable simultaneous disease diagnosis, treatment, and monitoring, representing an innovative approach to precision medicine. Luminescent transition metal complexes have emerged as promising alternatives to traditional organic fluorophores for optical imaging and theranostic applications, and they offer distinctive advantages such as high photostability, long emission lifetimes, and efficient generation of reactive oxygen species. In this Outlook, we highlight emerging trends in the designs and applications of luminescent transition metal complexes as optical imaging and theranostic agents, leveraging their unique photophysical and photochemical properties. We also discuss current challenges to the clinical applications of these complexes and outline potential directions to inspire the development of next-generation theranostics for advancing disease management and improving patient care.

## Graphical abstract

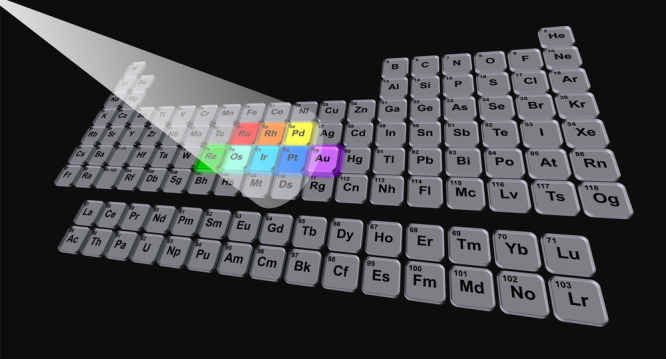

## Introduction

Fluorescence imaging is a noninvasive optical imaging technique using fluorescent probes for real-time visualization and tracking of cellular events with high spatial resolution, offering valuable insights into complex biological processes.^1–3^ The development of fluorogenic probes, which display fluorescence turn-on upon specific molecular interactions,^4–6^ has facilitated the detection, tracking, and quantification of biological species in their native contexts, highlighting their diverse roles in physiology and pathology.^7–11^ Fluorogenic probes targeting disease-associated biomarkers have enhanced disease diagnosis^12–16^ and enabled point-of-care device development for disease management.^17^ Additionally, advances in near-infrared (NIR) fluorescent probes have improved imaging depth and resolution, supporting *in vivo* diagnosis and fluorescence-guided surgery.^18–20^ Recent innovations in probe designs have led to fluorescent theranostics that combine therapeutic and diagnostic modalities in a single platform, paving the way for precision medicine.^21–23^

Luminescent transition metal complexes have manifested as promising alternatives to organic fluorophores for optical imaging^24–26^ and theranostic applications^27–29^ due to their rich photophysical and photochemical properties. Unlike organic fluorophores that emit from singlet excited states, many of these complexes exhibit intense phosphorescence from triplet excited states due to efficient intersystem crossing facilitated by the heavy metal center. This results in large Stokes’ shifts, minimizing self-quenching at high concentrations, and long emission lifetimes, supporting time-gated imaging techniques to eliminate short-lived autofluorescence for background-free imaging.^30^ Additionally, their long-lived triplet states facilitate energy transfer to molecular oxygen, which leads to (1) emission quenching, making them effective optical probes for detecting hypoxia;^31^ and (2) the generation of cytotoxic reactive oxygen species (ROS) such as singlet oxygen (^1^O_2_), rendering them potent photosensitizers (PSs) for photodynamic therapy (PDT).^32–34^ Furthermore, these complexes typically show high photostability under continuous irradiation, which is crucial for real-time imaging and effective phototherapy. Notably, the photophysical and photochemical behavior of these complexes are highly sensitive to their coordination environment and can be readily tuned through a judicious choice of metal centers and ligands, allowing their use in imaging,^24–26^ therapeutic,^32–34^ and theranostic^27–29^ applications.

Luminescent transition metal complexes have manifested as promising alternatives to organic fluorophores for optical imaging and theranostic applications due to their rich photophysical and photochemical properties.

In this Outlook, we highlight emerging trends in the designs and applications of luminescent transition metal complexes as optical imaging and theranostic agents. We also discuss current challenges and future prospects for their use in clinical practice, aiming to advance their development as effective theranostics and support their clinical translation for improving disease management and patient outcomes.

## Advancing Super-resolution and Time-Resolved Imaging

Fluorescence imaging is a powerful tool for visualizing cellular processes, but its resolution is often limited by optical diffraction and autofluorescence interference. Advances in super-resolution^35^ and time-resolved^36^ imaging techniques have revolutionized biological imaging, offering unprecedented spatial and temporal resolution. However, the rapid photobleaching and short emission lifetimes of conventional organic fluorophores limit their effectiveness. This underscores the need for more robust alternatives to fully harness these advanced techniques for deeper insights into complex biological systems.

### Super-Resolution Imaging

Super-resolution microscopy (SRM), such as structured illumination microscopy (SIM)^37^ and stimulated emission depletion (STED) microscopy,^38^ allows visualization of cellular structures at spatial resolution down to tens of nanometers,^39–41^ far surpassing the diffraction limit of conventional optical microscopy. Transition metal complexes have strong potential for development as SRM probes due to their high photostability, which enables prolonged imaging under the intense laser irradiation required for SRM.^42^ Dinuclear ruthenium­(II) (**1a**)^43,44^ and osmium­(II) (**1b**)^45^ complexes (Figure 1a), which can function as molecular light-switches for DNA, have been used as luminescent probes in SIM and STED microscopy to image nuclear DNA with enhanced resolution (Figure 1b). Using 3D-STED microscopy, the high-resolution visualization of the entire nucleus in three dimensions is achieved (Figure 1c), providing detailed morphological and functional insights. A mitochondria-targeting iridium­(III) complex (**2**) (Figure 1a) has been applied in SIM for super-resolution imaging of mitochondrial ultrastructures and real-time monitoring of mitochondrial dynamics in live cells.^46^ An iridium­(III) complex (**3**) (Figure 1a) that displays emission turn-on upon binding to tubulin has been utilized in STED microscopy to visualize microtubule networks in brain tissue, facilitating high-resolution imaging of neuronal subunits such as nascent neurites, growth cones, and spines (Figure 1d).^47^ Real-time tracking of the subcellular dynamics of a dinuclear platinum­(II) complex (**4**) (Figure 1a) under SIM reveals its light-induced escape from the autolysosomes to the nucleus, uncovering a novel transport pathway for platinum-based drugs.^48^

**Figure 1:**
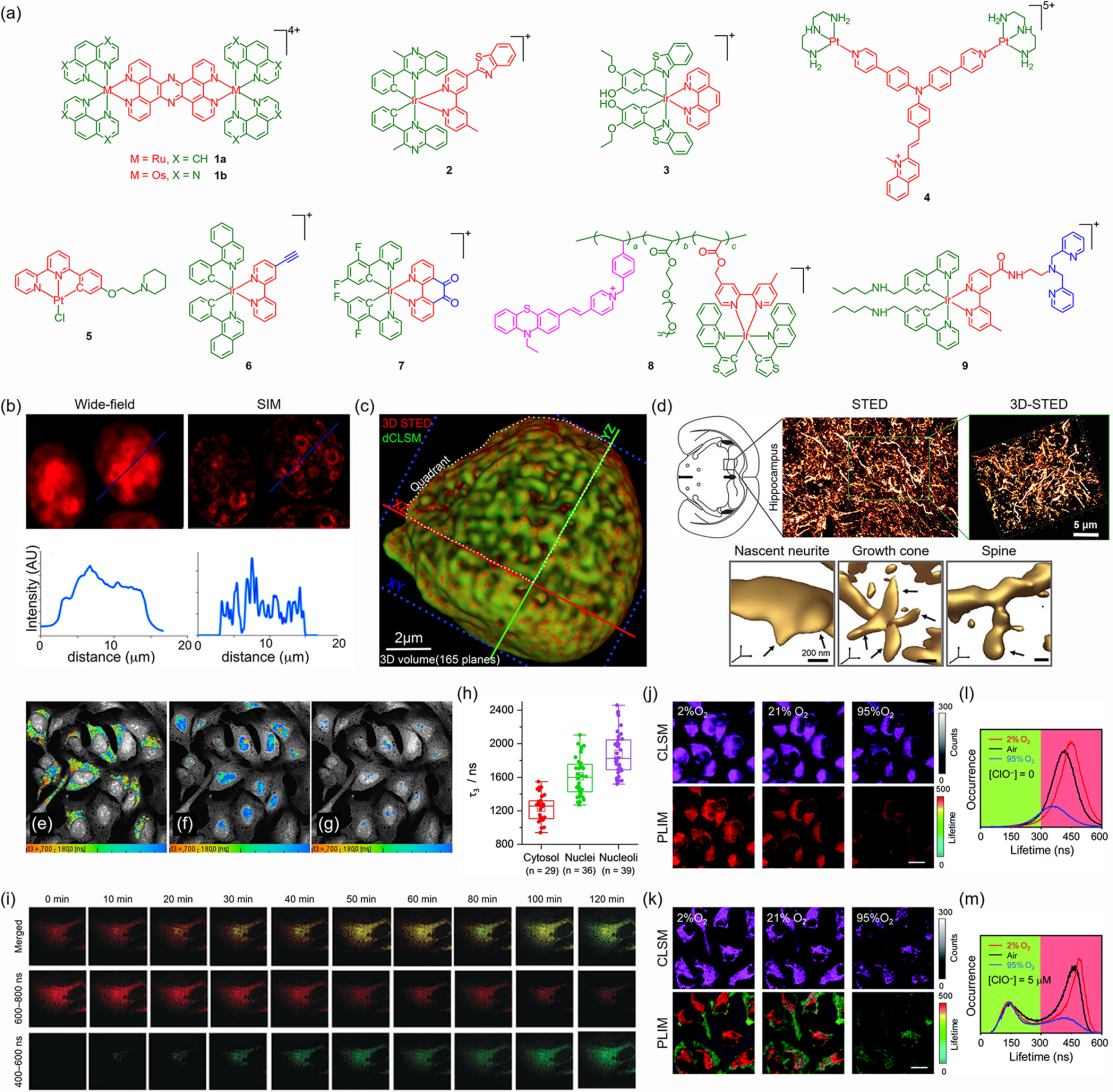
(a) Structures of complexes **1**–**9**. (b) Top: Wide-field fluorescence microscopy (left) and SIM (right) images of A2780 cells stained with complex **1a**. Bottom: Emission intensity profiles along the blue line in the respective images. (c) Super-resolution reconstruction of the entire nucleus, merging deconvoluted confocal laser-scanning microscopy (dCLSM) (green) and STED microscopy (red) images. Adapted with permission from ref 44. Copyright 2017 American Chemical Society. (d) Top: STED and 3D-STED microscopy images of brain hippocampus tissue stained with complex **3**. Bottom: Magnified regions show neuronal subunits (arrows). Adapted with permission from ref 47. Copyright 2020 Wiley-VCH. (e–g) PLIM images of U2OS cells stained with complex **5**, segmented into (e) cytosol, (f) nuclei, and (g) nucleoli. (h) Statistical analysis for the longest lifetime component (τ_3_) in segments shown in (e–g). Adapted with permission from ref 60. Available under a CC-BY 4.0 license. Copyright 2023 Wiley-VCH. (i) PLIM images of HeLa cells treated with l-azidohomoalanine and complex **6**, acquired at various time points postaddition of Cu_2_O NPs. Adapted with permission from ref 61. Copyright 2017 Wiley-VCH. (j and k) CLSM and PLIM images of complex **8**-stained HeLa cells (j) without and (k) with NaClO treatment and then incubated under different O_2_ conditions. Scale bars: 20 μm. (l and m) Lifetime distributions from PLIM images in (j) and (k). Adapted with permission from ref 63. Available under a CC-BY-NC 3.0 license. Copyright 2021 Royal Society of Chemistry.

### Time-Resolved Imaging

Time-resolved imaging techniques, such as photoluminescence lifetime imaging microscopy (PLIM),^36^ map emission lifetimes independently of probe concentration or excitation intensity, enabling quantitative analysis of biomolecular interactions and local environmental changes.^30,49^ Leveraging their environment-sensitive and long-lived emission, transition metal complexes have emerged as effective lifetime-responsive probes for imaging biological targets^50–54^ and microenvironments^55–59^ with negligible autofluorescence interference. A platinum­(II) complex (**5**) (Figure 1a) exhibits distinct emission lifetimes when bound to G-quadruplex DNA (1.42–1.90 μs) versus duplex DNA (*ca*. 0.75 μs), which allows selective visualization of these structures in cells via PLIM and reveals their predominant presence in the nucleoli (Figure 1e–h).^60^ Iridium­(III) alkyne (**6**)^61^ and phenanthrolinedione (**7**)^62^ complexes (Figure 1a) show substantial changes in lifetimes upon reaction with azide and α-angelica lactone, respectively. The incorporation of these abiotic moieties into the target proteins enables wash-free protein labeling and imaging by the complexes in live cells (Figure 1i). A dual-emissive polymeric probe (**8**) (Figure 1a) that integrates an O_2_-sensitive phosphorescent iridium­(III) complex and a hypochlorite (ClO^–^)-responsive fluorescent phenothiazine derivative has been developed for multiplex sensing.^63^ Although both luminophores emit at *ca*. 600 nm, their emission lifetimes differ by *ca*. 160 fold, allowing their emission response to be temporally resolved for the simultaneous imaging of O_2_ and ClO^–^ (Figure 1j–m). Building on our previous work on dual-emissive complexes,^64–66^ we have designed an iridium­(III) dipicolylamine complex (**9**) (Figure 1a) as the first molecular probe with orthogonal emission response to O_2_ and Cu^2+^, which enables the simultaneous detection of these species in live cells.^67^

## Near-Infrared-Emitting Probes for Cellular and *In Vivo* Imaging

Fluorescence imaging in the NIR spectral window (NIR-I: 700–900 nm; NIR-II: 1,000–1,700 nm) enables noninvasive deep-tissue imaging with high spatial resolution.^68–70^ Indocyanine green, an FDA-approved NIR fluorescent dye, is widely used for intraoperative imaging of sentinel lymph nodes, tumors, vascular structures, and tissue perfusion.^71^ Recently, OTL-38, a folate receptor-targeting NIR fluorescent probe, has also received FDA approval for fluorescence-guided surgery of ovarian and lung cancers.^72–74^ Several receptor-targeting NIR fluorescent probes are progressing through clinical trials for intraoperative cancer detection,^75^ including EMI-137 (c-Met),^76–78^ cRGD-ZW800-1 (integrins),^79,80^ cetuximab-IRDye800CW^81–84^ and panitumumab-IRDye800CW^85–87^ (epidermal growth factor receptor), and bevacizumab-IRDye800CW (vascular endothelial growth factor receptor).^88–91^ Despite these exciting developments, the small Stokes’ shifts and short fluorescence lifetimes of common organic fluorophores often limit imaging contrast in tissues with high autofluorescence background.

The development of NIR luminescent probes with long emission lifetimes enhances the visualization of deep biological tissues through time-resolved techniques, overcoming the limitations of traditional NIR fluorescence imaging.^92^ Major design strategies for NIR luminescent transition metal complexes primarily revolve around ligand engineering, including expanding π-conjugation length, incorporating polarizable heteroatoms, and introducing donor and acceptor moieties.^93^ Compared to coordinatively saturated octahedral d^6^ metal complexes, square-planar d^8^ metal complexes such as those of platinum­(II)^94^ and rhodium­(I)^95^ feature vacant axial coordination sites that allow metal–metal interactions between adjacent molecules, providing an additional dimension to manipulate their excited-state properties.^96^ For example, alkynylplatinum­(II) complexes **10** ^97^ and **11** ^98,99^ (Figure 2a) display enhanced triplet metal–metal-to-ligand charge-transfer (^3^MMLCT) emission upon supramolecular self-assembly in response to RNA and pH, respectively, via Pt···Pt and π–π interactions, which realizes specific imaging of the nucleoli and lysosomes. Selective cancer imaging has also been achieved using an alkynylplatinum­(II) complex featuring a histidine pendant (**12**) (Figure 2a), which exhibits ^3^MMLCT emission enhancement in the NIR region (Figure 2b) upon binding to sialic acids overexpressed on the cell surface (Figure 2c).^100^ An organoplatinum­(II) complex (**13**) (Figure 2a) that shows self-assembly-induced emission switching from green to red/NIR has been applied for live/dead cell differentiation through its distinctive emission response and unique lysosome-to-nucleus translocation (Figure 2d).^101^ Rhodium­(I) isocyanide complexes **14** ^102^ and **15** ^103^ (Figure 2a) have been formulated into nanoparticles (NPs) to enhance Rh···Rh interaction, which gives rise to intense NIR-I/II emission (Figure 2e) from 4dσ*­(Rh_*n*_) → 5pσ­(Rh_*n*_) (*n* ≥ 3) excited states for *in vivo* tumor imaging (Figure 2f). Related rhodium­(I) complexes (**16**) (Figure 2a), upon treatment with fetal bovine serum (FBS), have been developed as bright NIR-II luminescent probes.^104^ They have been utilized for *in vivo* tracking of single-macrophage dynamics (Figure 2g,h) and time-resolved imaging of abdominal organs (Figure 2i,j). Metallophilic interactions are also observed in d^10^ metal complexes; the Au···Au/Cu interaction in polymeric salts formed from cationic gold­(I) complexes and anionic gold­(I)/copper­(I) complexes, such as **17a** and **17b** (Figure 2a), respectively, promotes the population of a triplet ligand (anion)-to-ligand (cation) charge-transfer (^3^LLCT) emissive state,^105^ which has been harnessed for cancer-targeted imaging and PDT.^106^

The development of NIR luminescent probes with long emission lifetimes enhances the visualization of deep biological tissues through time-resolved techniques, overcoming the limitations of traditional NIR fluorescence imaging.

**Figure 2:**
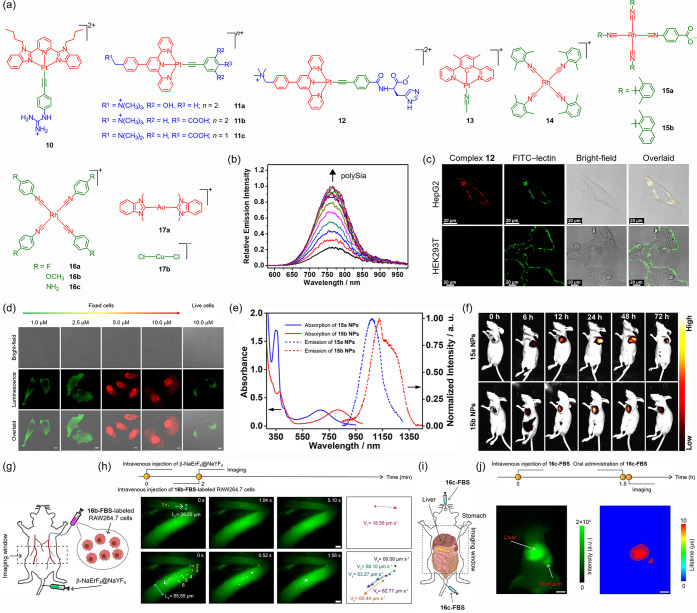
(a) Structures of complexes **10**–**17**. (b) Emission spectra of complex **12** upon the addition of polysialic acid (polySia) in Tris-HCl buffer. (c) CLSM images of HepG2 (top) and HEK293T cells (bottom) stained with complex **12** and fluorescein isothiocyanate (FITC)-conjugated lectin. Adapted with permission from ref 100. Available under a CC-BY 4.0 license. Copyright 2025 American Chemical Society. (d) CLSM images of fixed and live HeLa cells stained with complex **13** at different concentrations. Scale bars: 10 μm. Adapted with permission from ref 101. Copyright 2022 Wiley-VCH. (e) UV–vis absorption and normalized emission spectra of **15a NPs** and **15b NPs** in phosphate-buffered saline (PBS). (f) *In vivo* luminescence images of BEL-7404 tumor-bearing mice at various time points postintravenous injection of **15a NPs** or **15b NPs**. Adapted with permission from ref 103. Available under a CC-BY-NC 3.0 license. Copyright 2023 Royal Society of Chemistry. (g) Schematic depiction of *in vivo* tracking of macrophages. (h) Left: *In vivo* luminescence images of **16b**-**FBS**-labeled macrophages (magenta) in β-NaErF_4_@NaYF_4_-labeled blood vessels (green). L_1_ and L_2_ indicate the widths of the two blood vessels. Right: Trajectories and flowing velocities of labeled cells. Scale bars: 20 μm. (i) Schematic depiction of *in vivo* imaging of abdominal cavity. (j) *In vivo* luminescence (left) and time-resolved (right) images of mouse abdominal cavity postintravenous and oral administration of **16c**-**FBS**. Scale bars: 5 mm. Adapted with permission from ref 104. Copyright 2024 Wiley-VCH.

## Tailoring Precision for Disesase Diagnosis with Multilocked Probes

Luminogenic probes that display emission turn-on upon interaction with disease-specific biomarkers have advanced disease diagnosis and therapeutic development.^107^ Several protease-responsive fluorogenic probes have reached clinical evaluation for intraoperative tumor detection.^75^ For example, cathepsin-targeting LUM015 ^108,109^ and VGT-309 ^110,111^ have secured FDA approval or fast track designation for breast and lung cancer tissue visualization in patients undergoing lumpectomy and lung cancer surgery, respectively, while matrix metalloproteinase (MMP)-sensitive AVB-620 has completed phase II trials for malignant tissue detection during breast cancer surgery.^112,113^ However, most diseases lack a single definitive biomarker, and traditional single-locked probes may produce false positives due to moderate biomarker expression in healthy tissues, thereby restricting their clinical effectiveness. The development of multilocked probes that respond to multiple biomarkers offers a promising approach to overcoming these challenges.

### “AND”-Gated Probes

Multilocked probes incorporating an “AND” logic gate design have emerged, which require the simultaneous presence of distinct biomarkers for activation and thereby enhance detection specificity and diagnosis accuracy.^114–116^ An iridium­(III) poly­(ethylene glycol) (PEG) complex modified with an acid-sensitive imine linkage (**18**) (Figure 3a) has been designed as a tandem-locked nanoprobe that successively responds to acidity and hypoxia (Figure 3b), allowing specific imaging of tumors and metastatic lesions *in vivo*.^117^ A related iridium­(III) complex (**19**) (Figure 3a) with deeper NIR emission has enabled the ultrasensitive visualization of tumors as small as 260 × 290 μm (Figure 3c) and metastatic lesions down to 115 μm (Figure 3d).^118^ A ruthenium­(II) complex (**20**) (Figure 3a) exhibits emission turn-on upon specific reaction with formaldehyde (FA) in the acidic lysosomes of cancer cells, which allows the visualization of tumor-derived FA and *in vivo* monitoring of FA scavenging.^119^

**Figure 3:**
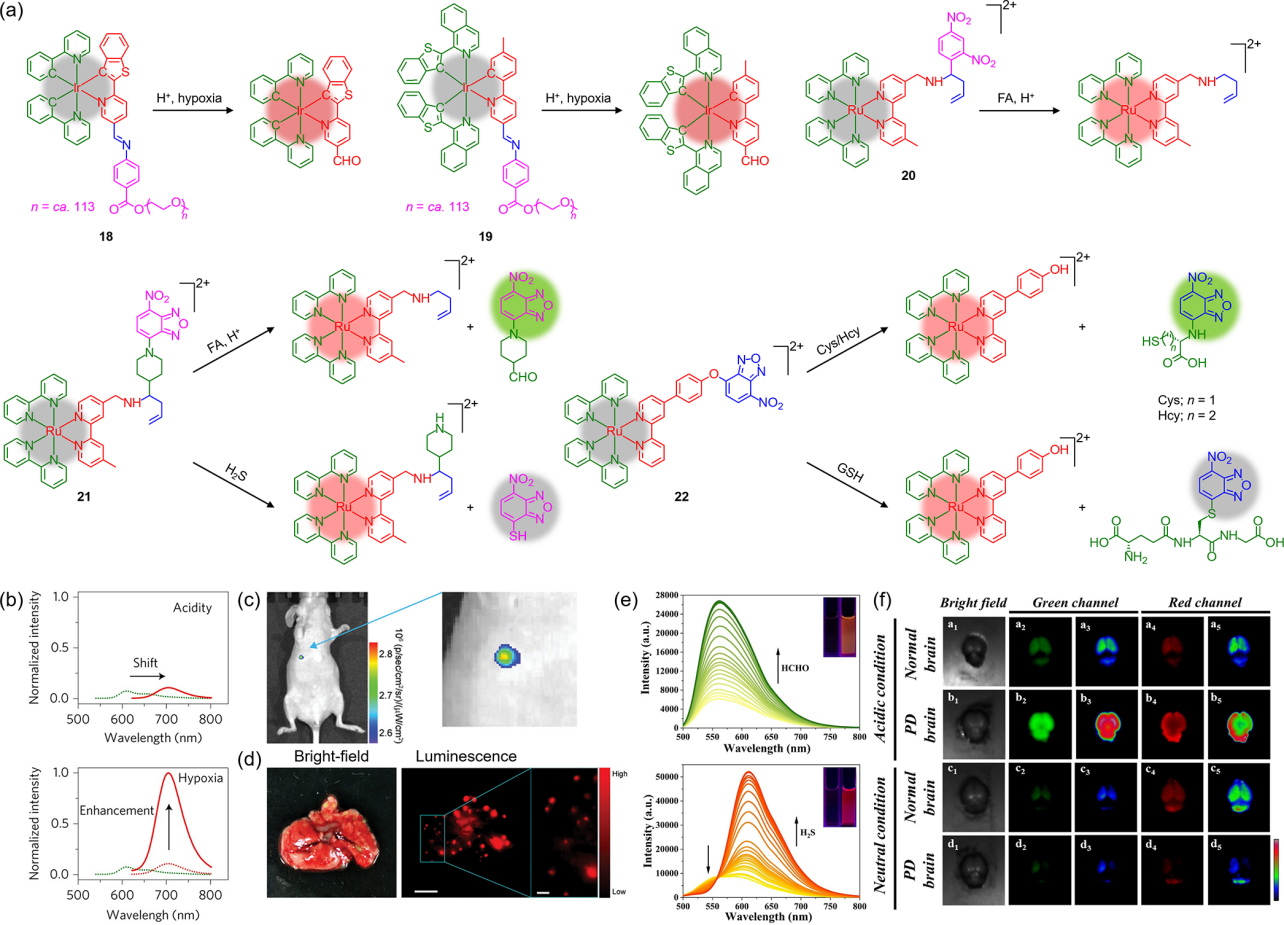
(a) Structures of complexes **18**–**22**. (b) Normalized emission spectra of complex **18** upon successive exposure to acidity and hypoxia. Adapted with permission from ref 117. Available under a CC-BY 4.0 license. Copyright 2017 Springer Nature. (c) *In vivo* NIR luminescence images of an orthotopic MDA-MB-231 tumor-bearing mouse at 24 h postintravenous injection of complex **19**. (d) *Ex vivo* bright-field and luminescence images of dissected lungs with metastatic nodules from an MDA-MB-231 tumor-bearing mouse at 24 h postinjection of complex **19**. Scale bars: 5 and 1 mm (magnified image). Adapted with permission from ref 118. Copyright 2023 Wiley-VCH. (e) Emission spectra of complex **21** in the presence of FA and H_2_S at different concentrations. (f) *Ex vivo* luminescence images of normal and PD mouse brains stained with complex **21**. Adapted with permission from ref 120. Copyright 2024 Elsevier.

### “OR”-Gated Probes

There is significant interest in “OR”-logic gated multilocked probes that show distinct emission signals in response to various biomarkers, which provide valuable insights into interconnected species and their roles in biological processes.^114–116^ A ruthenium­(II) nitrobenzoxadiazole (NBD) complex (**21**) (Figure 3a) displays distinct emission response to FA and hydrogen sulfide (H_2_S) (Figure 3e), which facilitates their discriminative visualization in Parkinson’s disease (PD) mouse brains (Figure 3f) and unveils, for the first time, a negative correlation between FA and H_2_S in PD pathogenesis.^120^ With a similar design strategy, a ruthenium­(II) NBD complex (**22**) (Figure 3a) has been developed for detecting total biothiols and distinguishing between cysteine (Cys)/homocysteine (Hcy) and glutathione (GSH).^121^ This complex serves as a powerful tool for elucidating the roles of these biothiols in cell biology, which is very challenging due to their structural and chemical similarities.

## Overcoming Oxygen Limitations in Photodynamic Therapy

Since the first clinical approval of Photofrin as a PS for bladder cancer treatment, PDT has developed as an effective cancer therapy with high spatiotemporal precision and minimal invasiveness.^122^ Several organic and metal-based PSs, including a ruthenium­(II) complex TLD1433,^123^ have been approved or are under clinical evaluation for PDT.^124^ However, most of these PSs rely on endogenous O_2_ for ^1^O_2_ generation, which limits their effectiveness in hypoxic solid tumors. Thus, it is crucial to develop new PDT agents with reduced or even no O_2_ dependence to meet clinical needs.

Targeted photoredox catalysis offers a promising approach for treating hypoxic tumors in clinical settings, with distinctive advantages over conventional PDT that primarily relies on O_2_ for ROS generation.

### Type I PSs for PDT

Compared to Type II PSs that exhibit high O_2_ dependence and consumption, Type I PSs generate ROS such as superoxide anion (O_2_^•–^) and hydroxyl (HO^•^) radicals via electron transfer to surrounding substrates, which facilitate ROS production even in hypoxic environments.^125^ Several transition metal complexes have been identified as effective Type I PSs with potent anticancer activity under hypoxic conditions.^126–135^ For example, a carbonic anhydrase IX-targeting rhenium­(I) oligothiophene complex (**23**) (Figure 4a) induces selective photocytotoxicity toward breast cancer cells under hypoxia through O_2_^•–^ generation, with ROS-mediated plasma membrane rupture resulting in pyroptosis and inducing adaptive immune response against both primary and distant tumors (Figure 4b–d).^136^ A palladium­(II) complex (**24**) (Figure 4a) self-assembles *in vivo*, via Pd···Pd interaction, into supramolecular nanostructures that show prolonged circulation and efficient tumor accumulation, which effectively destruct hypoxic melanoma tumors through O_2_^•–^ generation upon irradiation.^137^ Near-complete tumor ablation has been achieved *in vivo* using low doses of a ruthenium­(II) coumarin complex (**25**) (Figure 4a) that effectively generates O_2_^•–^ and HO^•^ upon deep red-light irradiation, overcoming the challenges of hypoxia and limited light penetration in oncological PDT.^138^

**Figure 4:**
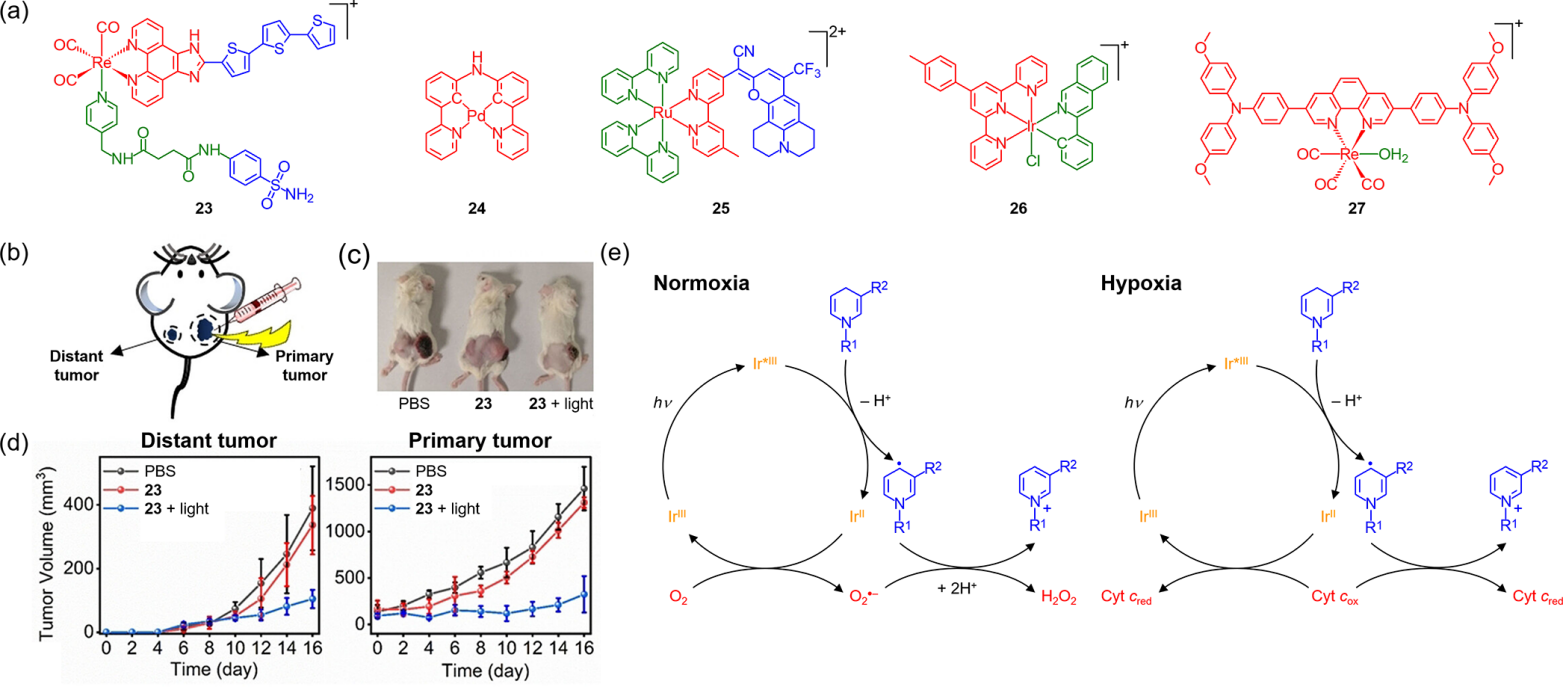
(a) Structures of complexes **23**–**27**. (b) Schematic depiction of *in vivo* PDT. (c) Photographs of bilateral 4T1 tumor-bearing mice after different treatments. (d) Tumor growth curves of distant and primary tumors in bilateral 4T1 tumor-bearing mice under different treatments. Adapted with permission from ref 136. Copyright 2022 Wiley-VCH. (e) Proposed catalytic cycles for NADH photooxidation under normoxia and hypoxia. Adapted with permission from ref 142. Available under a CC-BY 4.0 license. Copyright 2019 Springer Nature.

### Photoredox Catalysis

Although the reduced reliance of Type I PSs on endogenous O_2_ for ROS generation offers a partial solution to cancer treatment under hypoxic conditions, the short lifetimes and diffusion distances of ROS pose a clear challenge for the effective treatment of hypoxic, poorly vascularized tumors. The mean free paths of ROS such as ^1^O_2_ (<0.3 μm),^139^ O_2_^•–^ (<40 μm),^140^ and HO^•^ (<10 nm)^141^ are extremely short in biological environments, which means ROS must be generated in close proximity to their biological targets to be effective. This spatial constraint, combined with the limited O_2_ availability in the hypoxic tumor microenvironment (TME), reduces the effectiveness of traditional ROS-based PDT strategies against large solid tumors. Thus, exploring new O_2_-independent mechanisms is crucial to overcome hypoxia-induced resistance. A mitochondria-specific iridium­(III)-based photocatalyst (**26**) (Figure 4a) has been shown to kill hypoxic cancer cells via targeted photoredox catalysis.^142^ With its high excited-state reduction potential, the complex efficiently catalyzes the photooxidation of NADH to NAD^+^ via single electron transfer even under hypoxic conditions, by using cytochrome *c* (Cyt *c*) as the terminal electron acceptor (Figure 4e). The photocatalytic NADH/NAD^+^ conversion disrupts intracellular redox balance and induces effective cancer cell death under hypoxia, overcoming the O_2_ limitation of traditional PDT. This has stimulated the development of both metal-based^143–152^ and metal-free photocatalysts^153–162^ as phototherapeutic agents for treating hypoxic tumors. Recently, an electron donor–acceptor–donor-structured rhenium­(I) complex (**27**) (Figure 4a), which can catalyze the photooxidation of NADH to NAD^+^ and photoreduction of pyruvic acid to lactic acid while also generating ROS, has been applied for photoredox-mediated immunotherapy against hypoxic tumors.^163^ The resulting NADH depletion and ROS production disrupt redox balance, inducing ferroptosis and immunogenic cell death (ICD); while pyruvic acid depletion inhibits glycolysis and downregulates programmed death-ligand 1 expression, reinforcing the antitumor immune response. These studies highlight that targeted photoredox catalysis offers a promising approach for treating hypoxic tumors in clinical settings with distinctive advantages over conventional PDT that primarily relies on O_2_ for ROS generation.

## Activatable Phototheranostics for Targeted Photodynamic Therapy

While PDT presents significant potential for cancer treatment, conventional PSs often suffer from poor selectivity, leading to nonspecific binding to nontarget cells and accumulation in off-target organs (e.g., the liver). This off-target distribution compromises tumor targeting and therapeutic efficacy while increasing the risk of collateral damage to healthy tissues, which can result in acute toxicity and organ dysfunction. To improve tumor specificity, PSs have been conjugated to tumor-targeting moieties such as receptor-binding ligands, peptides, or antibodies that recognize specific biomarkers on cancer cells.^164^ These active targeting strategies enhance tumor accumulation and reduce off-target effects, thereby enabling more effective PDT. In general, a tumor-to-background ratio above 1.5 in fluorescence imaging is considered sufficient for distinguishing tumors from normal tissues in clinical settings,^165^ facilitating real-time image-guided PDT. However, the scarcity of universal biomarkers and the heterogeneity of their expression within and across tumors pose significant barriers to consistent and effective targeting.^166^ To address these challenges, there is growing interest in the development of activatable phototheranostics that display emission turn-on and controlled ROS generation in response to tumor-specific stimuli.^167,168^ This design approach can enhance tumor imaging and confine photocytotoxicity to malignant tissues, thereby improving the precision and safety of PDT.

Activatable phototheranostics that display emission turn-on and controlled ROS generation in response to tumor-specific stimuli can enhance tumor imaging and confine photocytotoxicity to malignant tissues, improving the precision and safety of PDT.

### TME-Responsive Phototheranostics

Transition metal complexes have been engineered as activatable phototheranostics that specifically target the distinct features of the TME. For example, an iridium­(III) ferrocene complex modified with an imine linkage (**28**) (Figure 5a) releases a photoactive iridium­(III) amine complex for ROS generation and a formyl ferrocene to induce ferroptosis in the acidic TME, leading to complete tumor ablation without recurrence (Figure 5b–e).^169^ An iridium­(III) *N*-alkylpyridinium complex (**29**) (Figure 5a) exhibits enhanced emission and ^1^O_2_ generation in response to elevated GSH levels, which allow selective imaging and photocytotoxicity in cancer cells (Figure 5f).^170^ A hypoxia-activatable theranostic prodrug (**30**) (Figure 5a) selectively eliminates hypoxic tumor cells and spheroids through targeting overexpressed reductases that cleave its azo linkage, releasing a photoactive ruthenium­(II) amine complex for ROS generation and NADH oxidation, along with an aniline mustard for DNA cross-linking.^171^ We have designed a peptide-based phototheranostic (**31**) (Figure 5a) that targets MMP-2/9, where enzymatic cleavage of the peptide linker disrupts Förster resonance energy transfer (FRET) from the iridium­(III) core to the QSY-7 quencher, resulting in emission enhancement and ^1^O_2_ generation for cancer-selective imaging and PDT.^172^

**Figure 5:**
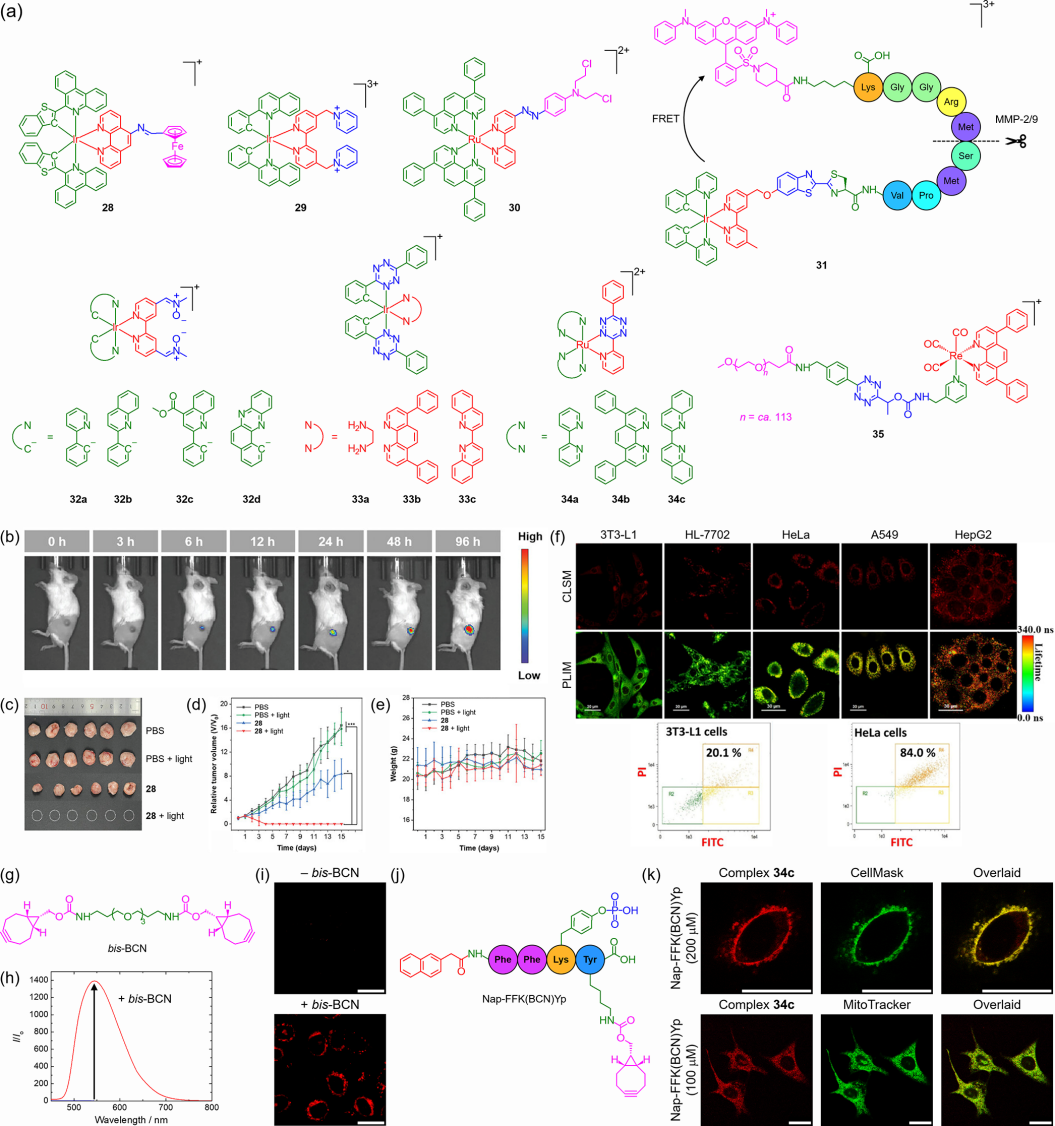
(a) Structures of complexes **28**–**35**. (b) *In vivo* luminescence images of 4T1 tumor-bearing mice at various time points postintratumoral injection of complex **28**. (c) Photographs of tumors dissected from 4T1 tumor-bearing mice after different treatments. (d) Tumor growth curves and (e) body weights of 4T1 tumor-bearing mice under different treatments. *p < 0.05 and ***p < 0.001. Adapted with permission from ref 169. Available under a CC-BY 4.0 license. Copyright 2025 Wiley-VCH. (f) Top: CLSM and PLIM images of noncancerous (3T3-L1 and HL-7702) and cancerous (HeLa, A549, and HepG2) cells stained with complex **29**. Scale bars: 30 μm. Bottom: Flow cytometric quantification of apoptosis in 3T3-L1 and HeLa cells treated with complex **29** and light irradiation. Adapted with permission from ref 170. Copyright 2019 Wiley-VCH. (g) Structure of *bis*-BCN. (h) Emission spectra of complex **33b** in H_2_O/DMSO (4:1, *v*/*v*) before (blue) and after (red) reaction with *bis*-BCN. (i) CLSM images of *bis*-BCN-untreated (top) or -pretreated (bottom) HeLa cells incubated with complex **33b**. Scale bars: 25 μm. Adapted with permission from ref 184. Copyright 2022 Wiley-VCH. (j) Structure of Nap-FFK­(BCN)­Yp. (k) CLSM images of HeLa cells treated with Nap-FFK­(BCN)­Yp at different concentrations, followed by incubation with complex **34c** and then CellMask Deep Red (top) or MitoTracker Deep Red FM (bottom). Scale bars: 25 μm. Adapted with permission from ref 185. Copyright 2023 Wiley-VCH.

### Bioorthogonally Activatable Phototheranostics

Bioorthogonal chemistry offers a powerful strategy to enhance the precision of PDT by using abiotic chemical functionalities as artificial receptors to direct the localization and activation of PSs via chemoselective reactions.^173^ This approach can minimize off-target activation and photocytotoxicity, especially when differences in endogenous biomarker expression between the tumor and normal tissues are subtle. Tetrazine^174^ and nitrone^175^ are known for their rapid and selective reactions with bicyclo[6.1.0]­non-4-yne (BCN). We have demonstrated that these bioorthogonal moieties can effectively modulate the photoactivity of transition metal complexes, enabling emission turn-on and ^1^O_2_ generation upon specific bioorthogonal reactions.^176–182^ Recently, we have developed iridium­(III) *bis*-nitrone (**32**)^183^ and *bis*-tetrazine (**33**)^184^ complexes (Figure 5a) that react rapidly with *bis*-BCN (Figure 5g) and show significantly enhanced emission (Figure 5h) and ^1^O_2_ generation, which allow selective imaging and photocytotoxicity toward *bis*-BCN-pretreated cells (Figure 5i). Additionally, we have exploited alkaline phosphatase-instructed self-assembly of a BCN-modified phosphopeptide, Nap-FFK­(BCN)­Yp (Figure 5j), to achieve targeted accumulation and activation of ruthenium­(II) tetrazine complexes (**34**) (Figure 5a) in cancer cells for selective imaging and PDT (Figure 5k).^185^ We have also utilized a “click-to-release” approach to regulate the photoactivity and cytotoxicity of a rhenium­(I) tetrazine–PEG complex (**35**) (Figure 5a), where the *trans*-cyclooctene-mediated cleavage of the tetrazine–carbamate linker releases a photoactive rhenium­(I) amine complex as a promising therapeutic that induces ICD.^186^

## Chemiluminescent Probes as Self-Illuminated Theranostics

Fluorescence imaging is useful for disease diagnosis and treatment monitoring, but its dependence on external light limits tissue penetration and introduces background noise from tissue autofluorescence. In contrast, chemiluminescence imaging eliminates the need for real-time photoexcitation, which facilitates deep-tissue imaging with superior signal-to-noise ratios.^187,188^ Thus, chemiluminescent transition metal complexes are expected to present intriguing possibilities for bioimaging and biomedical applications.^189^

### Chemiluminescence Imaging

Chemiluminescent transition metal complexes represent an attractive prospect for biomedical imaging. The classical ruthenium­(II) complex [Ru­(bpy)_3_]^2+^ (bpy = 2,2’-bipyridine) emits light upon redox triggering^190^ and has been applied in intraoperative imaging to aid surgical margin evaluation.^191^ Recently, an iridium­(III) Schiff base complex (**36**) (Figure 6a) that displays chemiluminescence upon reaction with ROS (Figure 6b) due to an unusual imine-to-amide conversion has been used as a chemiluminogen in chemiluminescent NPs for imaging ROS-related diseases (Figure 6c).^192^ A heterotrinuclear iridium­(III)–copper­(II) complex (**37**) (Figure 6a) that exhibits NIR chemiluminescence upon peroxynitrite (ONOO^–^)-mediated oxidation of the porphyrin ring has been utilized to construct chemiluminescent NPs for real-time *in vivo* thrombus imaging and image-guided thrombolysis (Figure 6d–f).^193^ Additionally, red/NIR luminescent iridium­(III) complexes conjugated with a green chemiluminescent phenoxy-1,2-dioxetane moiety (**38**) (Figure 6a) have been designed for excitation-free, ratiometric O_2_ imaging (Figure 6g) through chemiluminescence resonance energy transfer (CRET) from the chemi-excited benzoate to the iridium­(III) unit.^194^

**Figure 6:**
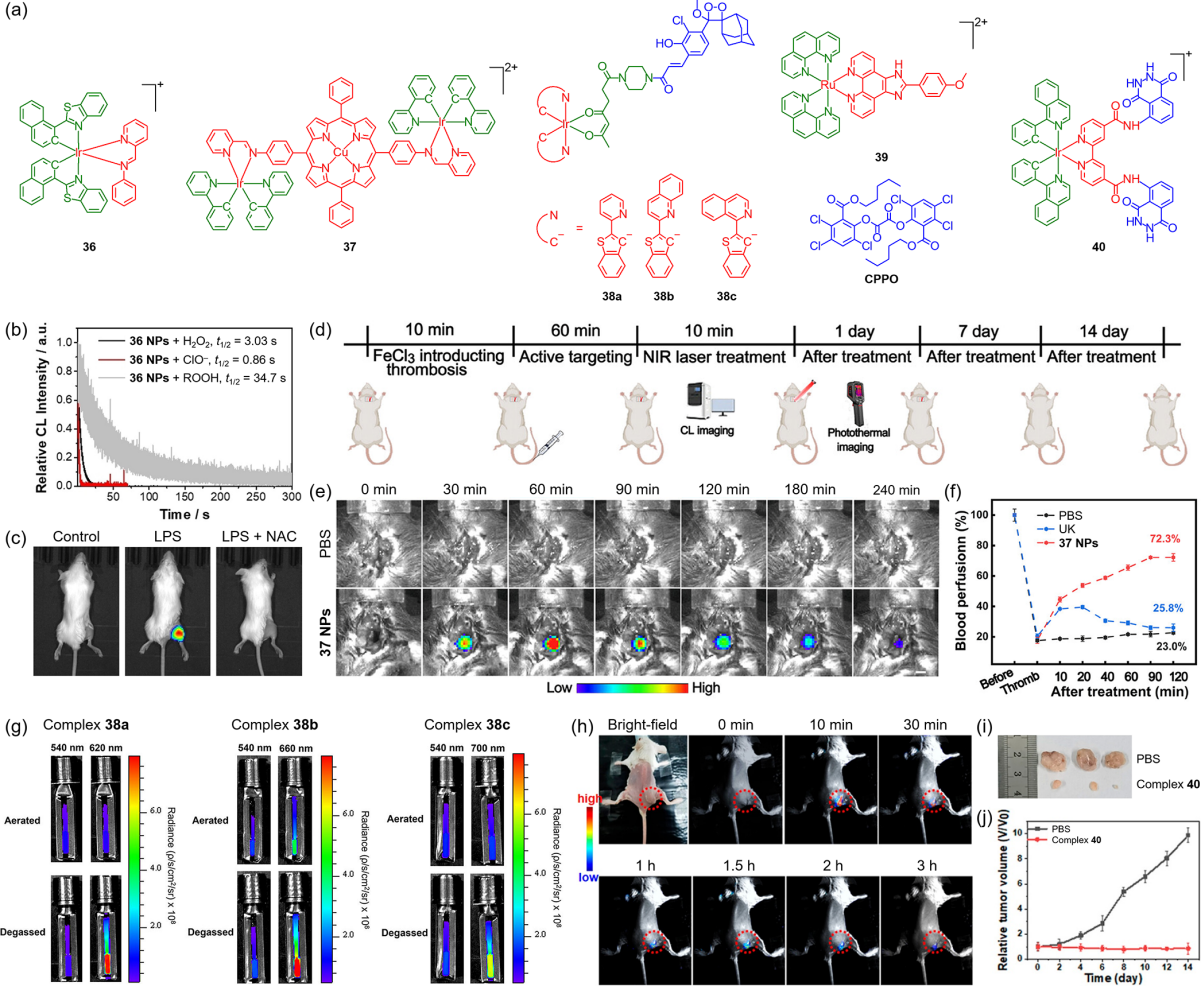
(a) Structures of complexes **36**–**40**. (b) Chemiluminescence decay curves of a mixture of **36 NPs** with H_2_O_2_ (black), ClO^–^ (red), and lipid peroxide (ROOH) (gray). (c) *In vivo* chemiluminescence images of lipopolysaccharide (LPS)-stimulated mice postintraarticular injection of **36 NPs** without and with pretreatment of *N*-acetylcysteine (NAC). Adapted with permission from ref 192. Copyright 2025 Wiley-VCH. (d) Schematic depiction of carotid artery thrombosis modeling and thrombolytic treatment. (e) *In vivo* chemiluminescence images of the thrombotic artery at different time points postintravenous injection of PBS and **37 NPs**. Scale bar: 5 mm. (f) Relative blood perfusion of the mouse carotid artery after FeCl_3_ treatment and different therapeutic treatments, including PBS, urokinase (UK), and **37 NPs**. Adapted with permission from ref 193. Available under a CC-BY 4.0 license. Copyright 2025 Wiley-VCH. (g) Chemiluminescence images of complexes **38a**–**38c** in PBS/DMSO (1:1, *v*/*v*) at different wavelengths under aerated and degassed conditions. Adapted with permission from ref 194. Copyright 2022 Wiley-VCH. (h) *In vivo* chemiluminescence images of a 4T1 tumor-bearing mouse at different time points postintratumoral injection of complex **40**. (i) Photographs of tumors dissected from 4T1 tumor-bearing mice after different treatments. (j) Tumor growth curves of 4T1 tumor-bearing mice under different treatments. Adapted with permission from ref 197. Available under a CC-BY 4.0 license. Copyright 2024 American Chemical Society.

### Self-Illuminated PDT

Chemiluminogens can act as molecular light sources to activate phototherapeutics, overcoming the light limitations of conventional phototherapies in treating deep-seated and metastatic tumors.^195^ A self-illuminated theranostic has been developed through coencapsulating a ruthenium­(II) complex (**39**) and bis­(2,4,5-trichloro-6-(pentyloxycarbonyl)­phenyl)­oxalate (CPPO) (Figure 6a) into liposomes.^196^ The reaction of CPPO with endogenous hydrogen peroxide (H_2_O_2_) in cancer cells excites the ruthenium­(II) complex via CRET, resulting in ^1^O_2_ generation and NADH oxidation. Notably, the chemi-excited NADH oxidation is accompanied by H_2_O_2_ generation, which permits self-amplified PDT for effective suppression of both primary and metastatic tumors. An iridium­(III) complex conjugated with two luminol moieties (**40**) (Figure 6a) has been designed as a unimolecular self-illuminated theranostic.^197^ The covalent linkage enhances CRET upon the hemoglobin-catalyzed luminol–H_2_O_2_ reaction, thereby facilitating the generation of iridium­(III)-based red emission and ROS for tumor imaging and PDT (Figure 6h–j), respectively.

Chemiluminescent transition metal complexes offer significant advantages as theranostics over their photoluminescent counterparts by eliminating the need for external light sources. However, their development is still in its infancy, facing challenges such as low brightness, short half-lives, and limited responsiveness to ROS. Phenoxy-1,2-dioxetanes offer a versatile scaffold for the development of novel chemiluminescent complexes, with tunable brightness^198^ and half-lives^199^ as well as high modularity for integrating various stimuli-responsive moieties^200^ to target a broad spectrum of disease biomarkers for both diagnosis and therapy.

## Conclusions

Luminescent transition metal complexes have demonstrated significant promise as optical imaging and theranostic agents due to their unique photophysical and photochemical properties. Their dual imaging and therapeutic capabilities facilitate simultaneous disease diagnosis, treatment, and monitoring, making them highly valuable for precision medicine. Despite encouraging results *in vitro* and in animal models, the clinical translation of these complexes for theranostic applications remains challenging. This is due to limited imaging contrast in deep tissues, reduced therapeutic efficacy in hypoxic environments, insufficient selectivity for diseased tissues, and suboptimal pharmacokinetics that hinder targeted accumulation and clearance. To advance their clinical adoption, future research should focus on innovative designs that integrate (1) NIR emission for high-resolution, deep-tissue imaging; (2) efficient Type I ROS generation or photoredox catalysis for hypoxia-tolerant phototherapy; and (3) multistimuli responsiveness for precise, disease-site-specific activation. Their broader clinical use will also necessitate optimization of pharmacokinetics to ensure effective tissue targeting, while enabling renal clearance to minimize off-target effects. Although structural refinement is often time-intensive, emerging technologies, such as artificial intelligence, offer powerful tools to accelerate the discovery and optimization of promising leads for clinical applications. We are confident that luminescent transition metal complexes will continue to play a central role in theranostics, with successful clinical adoption for disease diagnosis and treatment anticipated in the near future.

## References

[1] Zhang J., Campbell R. E., Ting A. Y., Tsien R. Y. (2002). Creating New Fluorescent
Probes for Cell Biology. Nat. Rev. Mol. Cell
Biol..

[2] Giepmans B.
N. G., Adams S. R., Ellisman M. H., Tsien R. Y. (2006). The Fluorescent
Toolbox for Assessing Protein Location and Function. Science.

[3] Xu W., Zeng Z., Jiang J.-H., Chang Y.-T., Yuan L. (2016). Discerning
the Chemistry in Individual Organelles with Small-Molecule Fluorescent
Probes. Angew. Chem., Int. Ed..

[4] Chan J., Dodani S. C., Chang C. J. (2012). Reaction-Based
Small-Molecule Fluorescent
Probes for Chemoselective Bioimaging. Nat. Chem..

[5] Yang Y., Zhao Q., Feng W., Li F. (2013). Luminescent Chemodosimeters
for Bioimaging. Chem. Rev..

[6] Bruemmer K. J., Crossley S. W. M., Chang C. J. (2020). Activity-Based
Sensing: A Synthetic
Methods Approach for Selective Molecular Imaging and Beyond. Angew. Chem., Int. Ed..

[7] Grover K., Koblova A., Pezacki A. T., Chang C. J., New E. J. (2024). Small-Molecule
Fluorescent Probes for Binding- and Activity-Based Sensing of Redox-Active
Biological Metals. Chem. Rev..

[8] Geng Y., Wang Z., Zhou J., Zhu M., Liu J., James T. D. (2023). Recent Progress in the Development
of Fluorescent Probes
for Imaging Pathological Oxidative Stress. Chem.
Soc. Rev..

[9] Niu L.-Y., Chen Y.-Z., Zheng H.-R., Wu L.-Z., Tung C.-H., Yang Q.-Z. (2015). Design Strategies
of Fluorescent Probes for Selective
Detection among Biothiols. Chem. Soc. Rev..

[10] Zhang J., Chai X., He X.-P., Kim H.-J., Yoon J., Tian H. (2019). Fluorogenic Probes
for Disease-Relevant Enzymes. Chem. Soc. Rev..

[11] Ma J., Sun R., Xia K., Xia Q., Liu Y., Zhang X. (2024). Design and
Application of Fluorescent Probes to Detect Cellular Physical Microenvironments. Chem. Rev..

[12] Wang X., Ding Q., Groleau R. R., Wu L., Mao Y., Che F., Kotova O., Scanlan E. M., Lewis S. E., Li P., Tang B., James T. D., Gunnlaugsson T. (2024). Fluorescent
Probes for Disease Diagnosis. Chem. Rev..

[13] Zhou J., Jangili P., Son S., Ji M. S., Won M., Kim J. S. (2020). Fluorescent Diagnostic
Probes in Neurodegenerative
Diseases. Adv. Mater..

[14] Huang J., Pu K. (2021). Near-Infrared Fluorescent Molecular Probes for Imaging and Diagnosis
of Nephro-Urological Diseases. Chem. Sci..

[15] Han H.-H., Tian H., Zang Y., Sedgwick A. C., Li J., Sessler J. L., He X.-P., James T. D. (2021). Small-Molecule Fluorescence-Based
Probes for Interrogating Major Organ Diseases. Chem. Soc. Rev..

[16] Wu L., Li Z., Wang K., Groleau R. R., Rong X., Liu X., Liu C., Lewis S. E., Zhu B., James T. D. (2025). Advances in Organic
Small Molecule-Based Fluorescent Probes for Precision Detection of
Liver Diseases: A Perspective on Emerging Trends and Challenges. J. Am. Chem. Soc..

[17] Zhan J., Cai Y., Cheng P., Zheng L., Pu K. (2025). Body Fluid Diagnostics
Using Activatable Optical Probes. Chem. Soc.
Rev..

[18] Hong G., Antaris A. L., Dai H. (2017). Near-Infrared
Fluorophores for Biomedical
Imaging. Nat. Biomed. Eng..

[19] He S., Song J., Qu J., Cheng Z. (2018). Crucial Breakthrough
of Second Near-Infrared Biological Window Fluorophores: Design and
Synthesis toward Multimodal Imaging and Theranostics. Chem. Soc. Rev..

[20] Lei Z., Zhang F. (2021). Molecular Engineering of NIR-II Fluorophores for Improved Biomedical
Detection. Angew. Chem., Int. Ed..

[21] Lee M. H., Sharma A., Chang M. J., Lee J., Son S., Sessler J. L., Kang C., Kim J. S. (2018). Fluorogenic
Reaction-Based
Prodrug Conjugates as Targeted Cancer Theranostics. Chem. Soc. Rev..

[22] Han H.-H., Wang H.-M., Jangili P., Li M., Wu L., Zang Y., Sedgwick A. C., Li J., He X.-P., James T. D., Kim J. S. (2023). The Design of Small-Molecule Prodrugs
and Activatable Phototherapeutics for Cancer Therapy. Chem. Soc. Rev..

[23] Sharma A., Verwilst P., Li M., Ma D., Singh N., Yoo J., Kim Y., Yang Y., Zhu J.-H., Huang H., Hu X.-L., He X.-P., Zeng L., James T. D., Peng X., Sessler J. L., Kim J. S. (2024). Theranostic Fluorescent
Probes. Chem. Rev..

[24] Zhao Q., Huang C., Li F. (2011). Phosphorescent
Heavy-Metal Complexes
for Bioimaging. Chem. Soc. Rev..

[25] Baggaley E., Weinstein J. A., Williams J. A. G. (2012). Lighting the Way to See inside the
Live Cell with Luminescent Transition Metal Complexes. Coord. Chem. Rev..

[26] Lee L. C.-C., Lo K. K.-W. (2024). Shining New Light on Biological Systems:
Luminescent
Transition Metal Complexes for Bioimaging and Biosensing Applications. Chem. Rev..

[27] Chen Y., Guan R., Zhang C., Huang J., Ji L., Chao H. (2016). Two-Photon Luminescent Metal Complexes for Bioimaging
and Cancer
Phototherapy. Coord. Chem. Rev..

[28] Tan C.-P., Zhong Y.-M., Ji L.-N., Mao Z.-W. (2021). Phosphorescent Metal
Complexes as Theranostic Anticancer Agents: Combining Imaging and
Therapy in a Single Molecule. Chem. Sci..

[29] Lee L. C.-C., Lo K. K.-W. (2022). Luminescent and
Photofunctional Transition Metal Complexes:
From Molecular Design to Diagnostic and Therapeutic Applications. J. Am. Chem. Soc..

[30] Zhang K. Y., Yu Q., Wei H., Liu S., Zhao Q., Huang W. (2018). Long-Lived
Emissive Probes for Time-Resolved Photoluminescence Bioimaging and
Biosensing. Chem. Rev..

[31] Yoshihara T., Hirakawa Y., Hosaka M., Nangaku M., Tobita S. (2017). Oxygen Imaging
of Living Cells and Tissues Using Luminescent Molecular Probes. J. Photochem. Photobiol. C.

[32] McKenzie L. K., Bryant H. E., Weinstein J. A. (2019). Transition
Metal Complexes as Photosensitisers
in One- and Two-Photon Photodynamic Therapy. Coord. Chem. Rev..

[33] Wu Y., Li S., Chen Y., He W., Guo Z. (2022). Recent Advances
in
Noble Metal Complex Based Photodynamic Therapy. Chem. Sci..

[34] Lee L. C.-C., Lo K. K.-W. (2024). Leveraging the
Photofunctions of Transition Metal Complexes
for the Design of Innovative Phototherapeutics. Small Methods.

[35] Schermelleh L., Ferrand A., Huser T., Eggeling C., Sauer M., Biehlmaier O., Drummen G. P. C. Super-Resolution
Microscopy Demystified. (2019). Nat. Cell
Biol..

[36] Torrado B., Pannunzio B., Malacrida L., Digman M. A. (2024). Fluorescence Lifetime
Imaging Microscopy. Nat. Rev. Methods Primers.

[37] Heintzmann R., Huser T. (2017). Super-Resolution Structured
Illumination Microscopy. Chem. Rev..

[38] Blom H., Widengren J. (2017). Stimulated
Emission Depletion Microscopy. Chem. Rev..

[39] Huang B., Babcock H., Zhuang X. (2010). Breaking the
Diffraction Barrier:
Super-Resolution Imaging of Cells. Cell.

[40] Sahl S. J., Hell S. W., Jakobs S. (2017). Fluorescence
Nanoscopy in Cell Biology. Nat. Rev. Mol. Cell
Biol..

[41] Sigal Y. M., Zhou R., Zhuang X. (2018). Visualizing and Discovering Cellular
Structures with Super-Resolution Microscopy. Science.

[42] Yusoh N. A., Gill M. R., Tian X. (2025). Advancing Super-Resolution Microscopy
with Metal Complexes: Functional Imaging Agents for Nanoscale Visualization. Chem. Soc. Rev..

[43] Gill M. R., Garcia-Lara J., Foster S. J., Smythe C., Battaglia G., Thomas J. A. (2009). A Ruthenium­(II) Polypyridyl Complex for Direct Imaging
of DNA Structure in Living Cells. Nat. Chem..

[44] Sreedharan S., Gill M. R., Garcia E., Saeed H. K., Robinson D., Byrne A., Cadby A., Keyes T. E., Smythe C., Pellett P., de la Serna J. B., Thomas J. A. (2017). Multimodal Super-Resolution
Optical Microscopy Using a Transition-Metal-Based Probe Provides Unprecedented
Capabilities for Imaging Both Nuclear Chromatin and Mitochondria. J. Am. Chem. Soc..

[45] Dröge F., Noakes F. F., Archer S. A., Sreedharan S., Raza A., Robertson C. C., MacNeil S., Haycock J. W., Carson H., Meijer A. J. H. M., Smythe C. G. W., de
la Serna J. B., Dietzek-Ivanšić B., Thomas J. A. (2021). A Dinuclear Osmium­(II) Complex Near-Infrared Nanoscopy
Probe for Nuclear DNA. J. Am. Chem. Soc..

[46] Chen Q., Jin C., Shao X., Guan R., Tian Z., Wang C., Liu F., Ling P., Guan J.-L., Ji L., Wang F., Chao H., Diao J. (2018). Super-Resolution Tracking of Mitochondrial
Dynamics with an Iridium­(III) Luminophore. Small.

[47] Tian X., De Pace C., Ruiz-Perez L., Chen B., Su R., Zhang M., Zhang R., Zhang Q., Wang Q., Zhou H., Wu J., Zhang Z., Tian Y., Battaglia G. (2020). A Cyclometalated
Iridium (III) Complex as a Microtubule
Probe for Correlative Super-Resolution Fluorescence and Electron Microscopy. Adv. Mater..

[48] Liu L.-Y., Fang H., Chen Q., Chan M. H.-Y., Ng M., Wang K.-N., Liu W., Tian Z., Diao J., Mao Z.-W., Yam V. W.-W. (2020). Multiple-Color
Platinum Complex with
Super-Large Stokes Shift for Super-Resolution Imaging of Autolysosome
Escape. Angew. Chem., Int. Ed..

[49] Berezin M. Y., Achilefu S. (2010). Fluorescence Lifetime
Measurements and Biological Imaging. Chem. Rev..

[50] You Y., Lee S., Kim T., Ohkubo K., Chae W.-S., Fukuzumi S., Jhon G.-J., Nam W., Lippard S. J. (2011). Phosphorescent Sensor
for Biological Mobile Zinc. J. Am. Chem. Soc..

[51] Xu W., Zhao X., Lv W., Yang H., Liu S., Liang H., Tu Z., Xu H., Qiao W., Zhao Q., Huang W. (2014). Rational Design of
Phosphorescent
Chemodosimeter for Reaction-Based One- and Two-Photon and Time-Resolved
Luminescent Imaging of Biothiols in Living Cells. Adv. Healthcare Mater..

[52] Chen Z., Yan P., Zou L., Zhao M., Jiang J., Liu S., Zhang K. Y., Huang W., Zhao Q. (2018). Using Ultrafast Responsive
Phosphorescent Nanoprobe to Visualize Elevated Peroxynitrite In Vitro
and In Vivo via Ratiometric and Time-Resolved Photoluminescence Imaging. Adv. Healthcare Mater..

[53] Wu Q., Zhang K. Y., Dai P., Zhu H., Wang Y., Song L., Wang L., Liu S., Zhao Q., Huang W. (2020). Bioorthogonal “Labeling after
Recognition” Affording
an FRET-Based Luminescent Probe for Detecting and Imaging Caspase-3
via Photoluminescence Lifetime Imaging. J. Am.
Chem. Soc..

[54] Wang W.-J., Mu X., Tan C.-P., Wang Y.-J., Zhang Y., Li G., Mao Z.-W. (2021). Induction and Monitoring of DNA Phase Separation in
Living Cells by a Light-Switching Ruthenium Complex. J. Am. Chem. Soc..

[55] Spencer J. A., Ferraro F., Roussakis E., Klein A., Wu J., Runnels J. M., Zaher W., Mortensen L. J., Alt C., Turcotte R., Yusuf R., Côté D., Vinogradov S. A., Scadden D. T., Lin C. P. (2014). Direct Measurement
of Local Oxygen Concentration in the Bone Marrow of Live Animals. Nature.

[56] Ma Y., Liang H., Zeng Y., Yang H., Ho C.-L., Xu W., Zhao Q., Huang W., Wong W.-Y. (2016). Phosphorescent Soft
Salt for Ratiometric and Lifetime Imaging of Intracellular pH Variations. Chem. Sci..

[57] Chen Z., Zhang K. Y., Tong X., Liu Y., Hu C., Liu S., Yu Q., Zhao Q., Huang W. (2016). Phosphorescent Polymeric
Thermometers for In Vitro and In Vivo Temperature Sensing with Minimized
Background Interference. Adv. Funct. Mater..

[58] Li X., Tong X., Yin Y., Yan H., Lu C., Huang W., Zhao Q. (2017). Using Highly Emissive
and Environmentally
Sensitive *o*-Carborane-Functionalized Metallophosphors
to Monitor Mitochondrial Polarity. Chem. Sci..

[59] Hao L., Li Z.-W., Zhang D.-Y., He L., Liu W., Yang J., Tan C.-P., Ji L.-N., Mao Z.-W. (2019). Monitoring
Mitochondrial Viscosity with Anticancer Phosphorescent Ir­(III) Complexes *via* Two-Photon Lifetime Imaging. Chem.
Sci..

[60] Berrones
Reyes J., Sherin P. S., Sarkar A., Kuimova M. K., Vilar R. (2023). Platinum­(II)-Based Optical Probes for Imaging Quadruplex DNA Structures
via Phosphorescence Lifetime Imaging Microscopy. Angew. Chem., Int. Ed..

[61] Wang J., Xue J., Yan Z., Zhang S., Qiao J., Zhang X. (2017). Photoluminescence
Lifetime Imaging of Synthesized Proteins in Living Cells Using an
Iridium–Alkyne Probe. Angew. Chem., Int.
Ed..

[62] Dai P., Luo C., Xu Z., Sun S., Tian Y., Zhang K. Y., Lo K. K.-W., Liu S., Huang W., Wang H., Zhao Q. (2025). Phosphorescent
Iridium­(III) Phenanthrolinedione Complexes as Lifetime-Responsive
Bioorthogonal Probes for Wash-Free Time-Resolved Bioimaging of Cellular
Labeling. Angew. Chem., Int. Ed..

[63] Wu Q., Dai P., Wang Y., Zhang J., Li M., Zhang K. Y., Liu S., Huang W., Zhao Q. (2021). Time-Resolved Analysis of Photoluminescence
at a Single Wavelength for Ratiometric and Multiplex Biosensing and
Bioimaging. Chem. Sci..

[64] Lo K. K.-W., Zhang K. Y., Leung S.-K., Tang M.-C. (2008). Exploitation of
the Dual-Emissive Properties of Cyclometalated Iridium­(III)–Polypyridine
Complexes in the Development of Luminescent Biological Probes. Angew. Chem., Int. Ed..

[65] Zhang K. Y., Liu H.-W., Tang M.-C., Choi A. W.-T., Zhu N., Wei X.-G., Lau K.-C., Lo K. K.-W. (2015). Dual-Emissive
Cyclometalated Iridium­(III) Polypyridine Complexes as Ratiometric
Biological Probes and Organelle-Selective Bioimaging Reagents. Inorg. Chem..

[66] Zhang K. Y., Gao P., Sun G., Zhang T., Li X., Liu S., Zhao Q., Lo K. K.-W., Huang W. (2018). Dual-Phosphorescent
Iridium­(III) Complexes Extending Oxygen Sensing from Hypoxia to Hyperoxia. J. Am. Chem. Soc..

[67] Zhu R., Dai P., Yang J., Zhou J., Zhang J., Zhang K. Y., Li Y., Liu S., Lo K. K.-W., Zhao Q. (2023). Dual-Emissive Iridium­(III)
Complexes as Phosphorescent Probes with Orthogonal Responses to Analyte
Binding and Oxygen Quenching. Angew. Chem.,
Int. Ed..

[68] Chen Y., Wang S., Zhang F. (2023). Near-Infrared Luminescence High-Contrast
In Vivo Biomedical Imaging. Nat. Rev. Bioeng..

[69] Wang F., Zhong Y., Bruns O., Liang Y., Dai H. (2024). In Vivo NIR-II
Fluorescence Imaging for Biology and Medicine. Nat. Photonics.

[70] Schmidt E. L., Ou Z., Ximendes E., Cui H., Keck C. H. C., Jaque D., Hong G. (2024). Near-Infrared II Fluorescence
Imaging. Nat.
Rev. Methods Primers.

[71] Vahrmeijer A. L., Hutteman M., van der
Vorst J. R., van de Velde C. J. H., Frangioni J. V. (2013). Image-Guided
Cancer Surgery Using Near-Infrared Fluorescence. Nat. Rev. Clin. Oncol..

[72] Hoogstins C. E. S., Tummers Q. R. J. G., Gaarenstroom K. N., de Kroon C. D., Trimbos J. B. M. Z., Bosse T., Smit V. T. H. B. M., Vuyk J., van de Velde C. J. H., Cohen A. F., Low P. S., Burggraaf J., Vahrmeijer A. L. (2016). A Novel Tumor-Specific Agent for
Intraoperative Near-Infrared Fluorescence Imaging: A Translational
Study in Healthy Volunteers and Patients with Ovarian Cancer. Clin. Cancer Res..

[73] Tanyi J. L., Randall L. M., Chambers S. K., Butler K. A., Winer I. S., Langstraat C. L., Han E. S., Vahrmeijer A. L., Chon H. S., Morgan M. A., Powell M. A., Tseng J. H., Lopez A. S., Wenham R. M. (2023). A Phase III Study of Pafolacianine
Injection (OTL38) for Intraoperative Imaging of Folate Receptor–Positive
Ovarian Cancer (Study 006). J. Clin. Oncol..

[74] Rice D., Singhal S., Niemeyer E., Sarkaria I., Martin L. W., Ebright M. I., Louie B. E., Lee T., Predina J. D. (2024). Intraoperative
Molecular Imaging with Pafolacianine in Resection of Occult Pulmonary
Malignancy in the ELUCIDATE Trial. Ann. Thorac.
Surg..

[75] Zhang R. R., Schroeder A. B., Grudzinski J. J., Rosenthal E. L., Warram J. M., Pinchuk A. N., Eliceiri K. W., Kuo J. S., Weichert J. P. (2017). Beyond the Margins:
Real-Time Detection of Cancer Using
Targeted Fluorophores. Nat. Rev. Clin. Oncol..

[76] Burggraaf J., Kamerling I. M. C., Gordon P. B., Schrier L., de Kam M. L., Kales A. J., Bendiksen R., Indrevoll B., Bjerke R. M., Moestue S. A., Yazdanfar S., Langers A. M. J., Swaerd-Nordmo M., Torheim G., Warren M. V., Morreau H., Voorneveld P. W., Buckle T., van Leeuwen F. W. B., Ødegårdstuen L.-I., Dalsgaard G. T., Healey A., Hardwick J. C. H. (2015). Detection of
Colorectal Polyps in
Humans Using an Intravenously Administered Fluorescent Peptide Targeted
against c-Met. Nat. Med..

[77] Armstrong G. R., Khot M. I., Portal C., West N. P., Perry S. L., Maisey T. I., Tiernan J. P., Hughes T. A., Tolan D. J., Jayne D. G. (2022). A Novel Fluorescent
c-Met Targeted Imaging Agent for
Intra-Operative Colonic Tumour Mapping: Translation from the Laboratory
into a Clinical Trial. Surg. Oncol..

[78] Jonker P. K. C., Metman M. J. H., Sondorp L. H. J., Sywak M. S., Gill A. J., Jansen L., Links T. P., van Diest P. J., van Ginhoven T. M., Löwik C. W. G.
M., Nguyen A. H., Coppes R. P., Robinson D. J., van Dam G. M., van Hemel B. M., Fehrmann R. S. N., Kruijff S. (2022). Intraoperative MET-Receptor Targeted
Fluorescent Imaging and Spectroscopy for Lymph Node Detection in Papillary
Thyroid Cancer: Novel Diagnostic Tools for More Selective Central
Lymph Node Compartment Dissection. Eur. J. Nucl.
Med. Mol. Imaging.

[79] Choi H. S., Gibbs S. L., Lee J. H., Kim S. H., Ashitate Y., Liu F., Hyun H., Park G., Xie Y., Bae S., Henary M., Frangioni J. V. (2013). Targeted
Zwitterionic Near-Infrared
Fluorophores for Improved Optical Imaging. Nat.
Biotechnol..

[80] de
Valk K. S., Deken M. M., Handgraaf H. J. M., Bhairosingh S. S., Bijlstra O. D., van Esdonk M. J., Terwisscha van Scheltinga A. G. T., Valentijn A. R. P. M., March T. L., Vuijk J., Peeters K. C. M. J., Holman F. A., Hilling D. E., Mieog J. S. D., Frangioni J. V., Burggraaf J., Vahrmeijer A. L. (2020). First-in-Human Assessment of cRGD-ZW800-1,
a Zwitterionic, Integrin-Targeted, Near-Infrared Fluorescent Peptide
in Colon Carcinoma. Clin. Cancer Res..

[81] Zinn K. R., Korb M., Samuel S., Warram J. M., Dion D., Killingsworth C., Fan J., Schoeb T., Strong T. V., Rosenthal E. L. (2015). IND-Directed
Safety and Biodistribution Study of Intravenously
Injected Cetuximab-IRDye800 in Cynomolgus Macaques. Mol. Imaging Biol..

[82] Rosenthal E. L., Warram J. M., de Boer E., Chung T. K., Korb M. L., Brandwein-Gensler M., Strong T. V., Schmalbach C. E., Morlandt A. B., Agarwal G., Hartman Y. E., Carroll W. R., Richman J. S., Clemons L. K., Nabell L. M., Zinn K. R. (2015). Safety
and Tumor Specificity of Cetuximab-IRDye800 for Surgical Navigation
in Head and Neck Cancer. Clin. Cancer Res..

[83] Miller S. E., Tummers W. S., Teraphongphom N., van den Berg N. S., Hasan A., Ertsey R. D., Nagpal S., Recht L. D., Plowey E. D., Vogel H., Harsh G. R., Grant G. A., Li G. H., Rosenthal E. L. (2018). First-in-Human
Intraoperative Near-Infrared
Fluorescence Imaging of Glioblastoma Using Cetuximab-IRDye800. J. Neurooncol..

[84] Tummers W. S., Miller S. E., Teraphongphom N. T., Gomez A., Steinberg I., Huland D. M., Hong S., Kothapalli S.-R., Hasan A., Ertsey R., Bonsing B. A., Vahrmeijer A. L., Swijnenburg R.-J, Longacre T. A., Fisher G. A., Gambhir S. S., Poultsides G. A., Rosenthal E. L. (2018). Intraoperative
Pancreatic Cancer
Detection Using Tumor-Specific Multimodality Molecular Imaging. Ann. Surg. Oncol..

[85] Heath C. H., Deep N. L., Sweeny L., Zinn K. R., Rosenthal E. L. (2012). Use of
Panitumumab-IRDye800 to Image Microscopic Head and Neck Cancer in
an Orthotopic Surgical Model. Ann. Surg. Oncol..

[86] Gao R. W., Teraphongphom N., de Boer E., van den Berg N. S., Divi V., Kaplan M. J., Oberhelman N. J., Hong S. S., Capes E., Colevas A. D., Warram J. M., Rosenthal E. L. (2018). Safety of Panitumumab-IRDye800CW
and Cetuximab-IRDye800CW
for Fluorescence-Guided Surgical Navigation in Head and Neck Cancers. Theranostics.

[87] Zhou Q., van den Berg N. S., Rosenthal E. L., Iv M., Zhang M., Vega Leonel J. C. M., Walters S., Nishio N., Granucci M., Raymundo R., Yi G., Vogel H., Cayrol R., Lee Y.-J., Lu G., Hom M., Kang W., Hayden Gephart M., Recht L., Nagpal S., Thomas R., Patel C., Grant G. A., Li G. (2021). EGFR-Targeted Intraoperative
Fluorescence Imaging Detects High-Grade Glioma with Panitumumab-IRDye800
in a Phase 1 Clinical Trial. Theranostics.

[88] Terwisscha
van Scheltinga A. G. T., van Dam G. M., Nagengast W. B., Ntziachristos V., Hollema H., Herek J. L., Schröder C. P., Kosterink J. G. W., Lub-de Hoog M. N., de Vries E. G. E. (2011). Intraoperative
Near-Infrared Fluorescence Tumor Imaging with Vascular Endothelial
Growth Factor and Human Epidermal Growth Factor Receptor 2 Targeting
Antibodies. J. Nucl. Med..

[89] Lamberts L. E., Koch M., de Jong J. S., Adams A. L. L., Glatz J., Kranendonk M. E. G., Terwisscha van Scheltinga A. G. T., Jansen L., de Vries J., Lub-de Hooge M. N., Schröder C. P., Jorritsma-Smit A., Linssen M. D., de Boer E., van der
Vegt B., Nagengast W. B., Elias S. G., Oliveira S., Witkamp A. J., Mali W. P. T. M., Van der Wall E., van Diest P. J., de Vries E. G. E., Ntziachristos V., van Dam G. M. (2017). Tumor-Specific Uptake
of Fluorescent Bevacizumab–IRDye800CW Microdosing in Patients
with Primary Breast Cancer: A Phase I Feasibility Study. Clin. Cancer Res..

[90] Steinkamp P. J., Pranger B. K., Li M.-F., Linssen M. D., Voskuil F. J., Been L. B., van Leeuwen B. L., Suurmeijer A. J. H., Nagengast W. B., Kruijff S., van Ginkel R. J., van Dam G. M. (2021). Fluorescence-Guided Visualization of Soft-Tissue Sarcomas
by Targeting Vascular Endothelial Growth Factor A: A Phase 1 Single-Center
Clinical Trial. J. Nucl. Med..

[91] Schmidt I., Vergeer R. A., Postma M. R., van den Berg G., Sterkenburg A. J., Korsten-Meijer A. G.
W., Feijen R. A., Kruijff S., van Beek A. P., den Dunnen W. F. A., Robinson D. J., van Dijk J. M. C., Nagengast W. B., Kuijlen J. M. A. (2025). Fluorescence Detection of Pituitary Neuroendocrine
Tumour during Endoscopic Transsphenoidal Surgery Using Bevacizumab-800CW:
A Non-Randomised, Non-Blinded, Single Centre Feasibility and Dose
Finding Trial [DEPARTURE Trial]. Eur. J. Nucl.
Med. Mol. Imaging.

[92] Chang B., Chen J., Bao J., Sun T., Cheng Z. (2023). Molecularly
Engineered Room-Temperature Phosphorescence for Biomedical Application:
From the Visible toward Second Near-Infrared Window. Chem. Rev..

[93] Xiang H., Cheng J., Ma X., Zhou X., Chruma J. J. (2013). Near-Infrared
Phosphorescence: Materials and Applications. Chem. Soc. Rev..

[94] Yam V. W.-W., Wong K. M.-C., Zhu N. (2002). Solvent-Induced Aggregation through
Metal···Metal/π···π Interactions:
Large Solvatochromism of Luminescent Organoplatinum­(II) Terpyridyl
Complexes. J. Am. Chem. Soc..

[95] Chen Y., Li K., Lloyd H. O., Lu W., Chui S. S.-Y., Che C.-M. (2010). Tetrakis­(arylisocyanide)
Rhodium­(I) Salts in Water: NIR Luminescent and Conductive Supramolecular
Polymeric Nanowires with Hierarchical Organization. Angew. Chem., Int. Ed..

[96] Yam V. W.-W., Au V. K.-M., Leung S. Y.-L. (2015). Light-Emitting Self-Assembled Materials
Based on d^8^ and d^10^ Transition Metal Complexes. Chem. Rev..

[97] Law A. S.-Y., Lee L. C.-C., Lo K. K.-W., Yam V. W.-W. (2021). Aggregation
and
Supramolecular Self-Assembly of Low-Energy Red Luminescent Alkynylplatinum­(II)
Complexes for RNA Detection, Nucleolus Imaging, and RNA Synthesis
Inhibitor Screening. J. Am. Chem. Soc..

[98] Chung C. Y.-S., Li S. P.-Y., Louie M.-W., Lo K. K.-W., Yam V. W.-W. (2013). Induced
Self-Assembly and Disassembly of Water-Soluble Alkynylplatinum­(II)
Terpyridyl Complexes with “Switchable” Near-Infrared
(NIR) Emission Modulated by Metal–Metal Interactions over Physiological
pH: Demonstration of pH-Responsive NIR Luminescent Probes in Cell-Imaging
Studies. Chem. Sci..

[99] Chung C. Y.-S., Li S. P.-Y., Lo K. K.-W., Yam V. W.-W. (2016). Synthesis and
Electrochemical, Photophysical, and Self-Assembly Studies on Water-Soluble
pH-Responsive Alkynylplatinum­(II) Terpyridine Complexes. Inorg. Chem..

[100] Guo J., Wong E. K.-H., Xu G.-X., Law A. S.-Y., Chan M. H.-Y., Lam J., Chen Z., Lo K. K.-W., Yam V. W.-W. (2025). Self-Assembly
of Alkynylplatinum­(II) Complexes for Sialic Acid Detection, and Differentiation
of Cancer Cells from Normal Cells. J. Am. Chem.
Soc..

[101] Li B., Wang Y., Chan M. H.-Y., Pan M., Li Y., Yam V. W.-W. (2022). Supramolecular
Assembly of Organoplatinum­(II) Complexes
for Subcellular Distribution and Cell Viability Monitoring with Differentiated
Imaging. Angew. Chem., Int. Ed..

[102] Wang J., Nie J.-J., Guo P., Yan Z., Yu B., Bu W. (2020). Rhodium­(I) Complex-Based Polymeric
Nanomicelles in
Water Exhibiting Coexistent Near-Infrared Phosphorescence Imaging
and Anticancer Activity In Vivo. J. Am. Chem.
Soc..

[103] Wei W., Wang J., Kang X., Li H., He Q., Chang G., Bu W. (2023). Synthesis, Supramolecular Aggregation,
and NIR-II Phosphorescence of Isocyanorhodium­(I) Zwitterions. Chem. Sci..

[104] Shi B., Zhang L., Yan K., Ming J., Chen Z.-H., Chen Y., He H., Zhang H., Wang L., Wang S., Zhang F. (2024). Efficient and Stable NIR-II Phosphorescence
of Metallophilic Molecular Oligomers for In Vivo Single-Cell Tracking
and Time-Resolved Imaging. Angew. Chem., Int.
Ed..

[105] Liu Q., Xie M., Chang X., Cao S., Zou C., Fu W.-F., Che C.-M., Chen Y., Lu W. (2018). Tunable Multicolor
Phosphorescence of Crystalline Polymeric Complex Salts with Metallophilic
Backbones. Angew. Chem., Int. Ed..

[106] Da X., Yu F.-H., Zhang C., Wang Z., Jian Y., Hou Y., Chen Y., Wang X., Zhou Q. (2022). A Bioorthogonal Assembly
Based on Metallophilic Interactions for Selective Imaging and PDT
Treatment of Cancer Cells. Inorg. Chem. Front..

[107] Dou W.-T., Han H.-H., Sedgwick A. C., Zhu G.-B., Zang Y., Yang X.-R., Yoon J., James T. D., Li J., He X.-P. (2022). Fluorescent Probes
for the Detection of Disease-Associated
Biomarkers. Sci. Bull..

[108] Whitley M. J., Cardona D. M., Lazarides A. L., Spasojevic I., Ferrer J. M., Cahill J., Lee C.-L., Snuderl M., Blazer D. G., Hwang E. S., Greenup R. A., Mosca P. J., Mito J. K., Cuneo K. C., Larrier N. A., O’Reilly E. K., Riedel R. F., Eward W. C., Strasfeld D. B., Fukumura D., Jain R. K., Lee W. D., Griffith L. G., Bawendi M. G., Kirsch D. G., Brigman B. E. (2016). A Mouse-Human
Phase 1 Co-Clinical Trial of a Protease-Activated Fluorescent Probe
for Imaging Cancer. Sci. Trans. Med..

[109] Smith B. L., Hunt K. K., Carr D., Blumencranz P. W., Hwang E. S., Gadd M. A., Stone K., Dyess D. L., Dodge D., Valente S., Dekhne N., Clark P., Lee M. C., Samiian L., Lesnikoski B.-A., Clark L., Smith K. P., Chang M., Harris D. K., Schlossberg B., Ferrer J., Wapnir I. L. (2023). Intraoperative Fluorescence
Guidance for Breast Cancer Lumpectomy Surgery. NEJM Evid..

[110] Suurs F. V., Qiu S.-Q., Yim J. J., Schröder C. P., Timmer-Bosscha H., Bensen E. S., Santini J. T., de Vries E. G. E., Bogyo M., van Dam G. M. (2020). Fluorescent
Image-Guided Surgery in Breast Cancer by Intravenous Application of
a Quenched Fluorescence Activity-Based Probe for Cysteine Cathepsins
in a Syngeneic Mouse Model. EJNMMI Res..

[111] Bou-Samra P., Kennedy G. T., Chang A., Guo E., Azari F. S., Din A., Santini J. T., Bensen E. S., Singhal S. (2025). Phase 2 Clinical Trial of VGT-309
for Intraoperative Molecular Imaging during Pulmonary Resection. Ann. Thorac. Surg..

[112] Miampamba M., Liu J., Harootunian A., Gale A. J., Baird S., Chen S. L., Nguyen Q. T., Tsien R. Y., González J. E. (2017). Sensitive *In Vivo* Visualization of Breast Cancer Using Ratiometric Protease-Activatable
Fluorescent Imaging Agent, AVB-620. Theranostics.

[113] Unkart J. T., Chen S. L., Wapnir I. L., González J. E., Harootunian A., Wallace A. M. (2017). Intraoperative Tumor
Detection Using
a Ratiometric Activatable Fluorescent Peptide: A First-in-Human Phase
1 Study. Ann. Surg. Oncol..

[114] Wu L., Huang J., Pu K., James T. D. (2021). Dual-Locked Spectroscopic
Probes for Sensing and Therapy. Nat. Rev. Chem..

[115] Xue S.-S., Li Y., Pan W., Li N., Tang B. (2023). Multi-Stimuli-Responsive Molecular Fluorescent Probes
for Bioapplications. Chem. Commun..

[116] Cheng P., Pu K. (2024). Enzyme-Responsive,
Multi-Lock Optical
Probes for Molecular Imaging and Disease Theranostics. Chem. Soc. Rev..

[117] Zheng X., Mao H., Huo D., Wu W., Liu B., Jiang X. (2017). Successively
Activatable Ultrasensitive Probe for Imaging
Tumour Acidity and Hypoxia. Nat. Biomed. Eng..

[118] Zhou S., Jiang L., Li C., Mao H., Jiang C., Wang Z., Zheng X., Jiang X. (2023). Acid and Hypoxia
Tandem-Activatable Deep Near-Infrared Nanoprobe for Two-Step Signal
Amplification and Early Detection of Cancer. Adv. Mater..

[119] Liu C., Zhang R., Zhang W., Liu J., Wang Y.-L., Du Z., Song B., Xu Z. P., Yuan J. (2019). “Dual-Key-and-Lock”
Ruthenium Complex Probe for Lysosomal Formaldehyde in Cancer Cells
and Tumors. J. Am. Chem. Soc..

[120] Ren J., Zhang W., Gao X., Song B., Yuan J. (2024). Unveiling
the Negative Correlation of Formaldehyde and Hydrogen Sulfide in Parkinson’s
Disease Models Using a Dual-Responsive Ruthenium­(II) Complex Probe. Chem. Eng. J..

[121] Liu C., Liu J., Zhang W., Wang Y.-L., Liu Q., Song B., Yuan J., Zhang R. (2020). “Two Birds with
One Stone” Ruthenium­(II) Complex Probe for Biothiols Discrimination
and Detection In Vitro and In Vivo. Adv. Sci..

[122] Dolmans D. E. J. G. J., Fukumura D., Jain R. K. (2003). Photodynamic
Therapy
for Cancer. Nat. Rev. Cancer.

[123] Monro S., Colón K. L., Yin H., Roque J., Konda P., Gujar S., Thummel R. P., Lilge L., Cameron C. G., McFarland S. A. (2019). Transition
Metal Complexes and Photodynamic Therapy from a Tumor-Centered Approach:
Challenges, Opportunities, and Highlights from the Development of
TLD1433. Chem. Rev..

[124] Karges J. (2022). Clinical Development of Metal Complexes
as Photosensitizers
for Photodynamic Therapy of Cancer. Angew. Chem.,
Int. Ed..

[125] Chen D., Xu Q., Wang W., Shao J., Huang W., Dong X. (2021). Type I Photosensitizers Revitalizing
Photodynamic Oncotherapy. Small.

[126] Lv Z., Wei H., Li Q., Su X., Liu S., Zhang K. Y., Lv W., Zhao Q., Li X., Huang W. (2018). Achieving Efficient Photodynamic Therapy under Both
Normoxia and
Hypoxia Using Cyclometalated Ru­(II) Photosensitizer through Type I
Photochemical Process. Chem. Sci..

[127] Novohradsky V., Rovira A., Hally C., Galindo A., Vigueras G., Gandioso A., Svitelova M., Bresolí-Obach R., Kostrhunova H., Markova L., Kasparkova J., Nonell S., Ruiz J., Brabec V., Marchán V. (2019). Towards Novel
Photodynamic Anticancer Agents Generating Superoxide Anion Radicals:
A Cyclometalated Ir^III^ Complex Conjugated to a Far-Red
Emitting Coumarin. Angew. Chem., Int. Ed..

[128] Roque J. A., Barrett P. C., Cole H. D., Lifshits L. M., Shi G., Monro S., von Dohlen D., Kim S., Russo N., Deep G., Cameron C. G., Alberto M. E., McFarland S. A. (2020). Breaking
the Barrier: An Osmium Photosensitizer with
Unprecedented Hypoxic Phototoxicity for Real World Photodynamic Therapy. Chem. Sci..

[129] Yuan H., Han Z., Chen Y., Qi F., Fang H., Guo Z., Zhang S., He W. (2021). Ferroptosis
Photoinduced by New Cyclometalated Iridium­(III) Complexes and Its
Synergism with Apoptosis in Tumor Cell Inhibition. Angew. Chem., Int. Ed..

[130] Ling Y.-Y., Wang W.-J., Hao L., Wu X.-W., Liang J.-H., Zhang H., Mao Z.-W., Tan C.-P. (2022). Self-Amplifying
Iridium­(III) Photosensitizer for Ferroptosis-Mediated Immunotherapy
against Transferrin Receptor-Overexpressing Cancer. Small.

[131] Ortega-Forte E., Rovira A., López-Corrales M., Hernández-García A., Ballester F. J., Izquierdo-García E., Jordà-Redondo M., Bosch M., Nonell S., Santana M. D., Ruiz J., Marchán V., Gasser G. (2023). A Near-Infrared Light-Activatable
Ru­(II)-Coumarin Photosensitizer Active under Hypoxic Conditions. Chem. Sci..

[132] Qi F., Yuan H., Chen Y., Peng X.-X., Wu Y., He W., Guo Z. (2023). Type I Photoreaction and Photoinduced Ferroptosis by
a Ru­(II) Complex to Overcome Tumor Hypoxia in Photodynamic Therapy. CCS Chem..

[133] Zhou J.-Y., Shen Q.-H., Hong X.-J., Zhang W.-Y., Su Q., Li W.-G., Cheng B., Tan C.-P., Wu T. (2023). Synergization
of an Endoplasmic Reticulum-Targeted Iridium­(III) Photosensitizer
with PD-L1 Inhibitor for Oral Squamous Cell Carcinoma Immunotherapy. Chem. Eng. J..

[134] Zhang Z., Wei Z., Guo J., Lyu J., Wang B., Wang G., Wang C., Zhou L., Yuan Z., Xing G., Wu C., Zhang X. (2024). Metallopolymer
Strategy to Explore Hypoxic Active Narrow-Bandgap Photosensitizers
for Effective Cancer Photodynamic Therapy. Nat.
Commun..

[135] Ren Q., Wang H., Li D., Dao A., Luo J., Wang D., Zhang P., Huang H. (2024). An Electron
Donor–Acceptor
Structured Rhenium­(I) Complex Photo-Sensitizer Evokes Mutually Reinforcing
“Closed-Loop” Ferroptosis and Immunotherapy. Adv. Healthcare Mater..

[136] Su X., Wang W.-J., Cao Q., Zhang H., Liu B., Ling Y., Zhou X., Mao Z.-W. (2022). A Carbonic Anhydrase
IX (CAIX)-Anchored Rhenium­(I) Photosensitizer Evokes Pyroptosis for
Enhanced Anti-Tumor Immunity. Angew. Chem.,
Int. Ed..

[137] Zhou X.-Q., Wang P., Ramu V., Zhang L., Jiang S., Li X., Abyar S., Papadopoulou P., Shao Y., Bretin L., Siegler M. A., Buda F., Kros A., Fan J., Peng X., Sun W., Bonnet S. (2023). In Vivo Metallophilic Self-Assembly of a Light-Activated
Anticancer Drug. Nat. Chem..

[138] Abad-Montero D., Gandioso A., Izquierdo-García E., Chumillas S., Rovira A., Bosch M., Jordà-Redondo M., Castaño D., Bonelli J., Novikov V. V., Deyà A., Hernández J. L., Galino J., Alberto M. E., Francés-Monerris A., Nonell S., Gasser G., Marchán V. (2025). Ruthenium­(II)
Polypyridyl Complexes Containing COUBPY Ligands as Potent Photosensitizers
for the Efficient Phototherapy of Hypoxic Tumors. J. Am. Chem. Soc..

[139] Skovsen E., Snyder J. W., Lambert J. D. C., Ogilby P. R. (2005). Lifetime
and Diffusion of Singlet Oxygen in a Cell. J.
Phys. Chem. B.

[140] Saran M., Bors W. (1994). Signalling by O_2_
^–•^ and NO^•^: How Far Can Either Radical, or Any Specific
Reaction Product, Transmit a Message under In Vivo Conditions?. Chem. Biol. Interact..

[141] Pryor W. A. (1986). Oxy-Radicals and Related Species: Their Formation,
Lifetimes, and Reactions. Annu. Rev. Physiol..

[142] Huang H., Banerjee S., Qiu K., Zhang P., Blacque O., Malcomson T., Paterson M. J., Clarkson G. J., Staniforth M., Stavros V. G., Gasser G., Chao H., Sadler P. J. (2019). Targeted
Photoredox Catalysis in Cancer Cells. Nat. Chem..

[143] Huang C., Liang C., Sadhukhan T., Banerjee S., Fan Z., Li T., Zhu Z., Zhang P., Raghavachari K., Huang H. (2021). In-Vitro and In-Vivo
Photocatalytic Cancer Therapy with Biocompatible Iridium­(III) Photocatalysts. Angew. Chem., Int. Ed..

[144] Fan Z., Rong Y., Sadhukhan T., Liang S., Li W., Yuan Z., Zhu Z., Guo S., Ji S., Wang J., Kushwaha R., Banerjee S., Raghavachari K., Huang H. (2022). Single-Cell Quantification of a Highly Biocompatible Dinuclear Iridium­(III)
Complex for Photocatalytic Cancer Therapy. Angew.
Chem., Int. Ed..

[145] Lu N., Deng Z., Gao J., Liang C., Xia H., Zhang P. (2022). An Osmium-Peroxo Complex for Photoactive Therapy of Hypoxic Tumors. Nat. Commun..

[146] Wei S., Liang H., Dao A., Xie Y., Cao F., Ren Q., Yadav A. K., Kushwaha R., Mandal A. A., Banerjee S., Zhang P., Ji S., Huang H. (2023). Perturbing Tumor Cell
Metabolism with a Ru­(II) Photo-Redox Catalyst to Reverse the Multidrug
Resistance of Lung Cancer. Sci. China Chem..

[147] Zhang C., Huang J., Guo X., Da X., Dai Z., Hassan M., Yu Y., Wang X., Zhou Q. (2023). NIR Light-Driven
Photocatalytic NAD­(P)H Oxidation and H_2_O_2_ Generation *In Situ* for Enhanced Chemodynamic Therapy and Immune Response. Nano Today.

[148] Shee M., Zhang D., Banerjee M., Roy S., Pal B., Anoop A., Yuan Y., Singh N. D. P. (2023). Interrogating
Bioinspired ESIPT/PCET-Based Ir­(III)-Complexes as Organelle-Targeted
Phototherapeutics: A Redox-Catalysis under Hypoxia to Evoke Synergistic
Ferroptosis/Apoptosis. Chem. Sci..

[149] Zhang J., Ma J., Zhang S., Lou X., Ding Y., Li Y., Xu M., Xie X., Jiao X., Dou X., Wang X., Tang B. (2023). Exploration
of Thermally Activated Delayed Fluorescence (TADF)-Based Photoredox
Catalyst to Establish the Mechanisms of Action for Photodynamic Therapy. ACS Nano.

[150] Xu Y., Chau C. V., Lee J., Sedgwick A. C., Yu L., Li M., Peng X., Kim J. S., Sessler J. L. (2024). Lutetium Texaphyrin:
A Photocatalyst that Triggers Pyroptosis via Biomolecular Photoredox
Catalysis. Proc. Natl. Acad. Sci. U.S.A..

[151] Dao A., Chen S., Pan L., Ren Q., Wang X., Wu H., Gong Q., Chen Z., Ji S., Ru J., Zhu H., Liang C., Zhang P., Xia H., Huang H. (2024). A 700 nm LED
Light Activated Ru­(II) Complex Destroys Tumor Cytoskeleton via Photosensitization
and Photocatalysis. Adv. Healthcare Mater..

[152] Shee M., Schleisiek J., Maity N., Das G., Montesdeoca N., Ha-Thi M.-H., Gore K. R., Karges J., Singh N. D. P. (2025). Exploring
Excited-State Intramolecular Proton-Coupled
Electron Transfer in Dinuclear Ir­(III)-Complex via Covalently Tagged
Hydroquinone: Phototherapy through Futile Redox Cycling. Small.

[153] Zhu Z., Wei L., Yadav A. K., Fan Z., Kumar A., Miao M., Banerjee S., Huang H. (2023). Cyanine-Functionalized
2,2’-Bipyridine Compounds for Photocatalytic Cancer Therapy. J. Org. Chem..

[154] Li M., Xu Y., Pu Z., Xiong T., Huang H., Long S., Son S., Yu L., Singh N., Tong Y., Sessler J. L., Peng X., Kim J. S. (2022). Photoredox
Catalysis May Be a General Mechanism in Photodynamic Therapy. Proc. Natl. Acad. Sci. U.S.A..

[155] Li M., Gebremedhin K. H., Ma D., Pu Z., Xiong T., Xu Y., Kim J. S., Peng X. (2022). Conditionally
Activatable Photoredox Catalysis in Living Systems. J. Am. Chem. Soc..

[156] Teng K.-X., Niu L.-Y., Xie N., Yang Q.-Z. (2022). Supramolecular
Photodynamic Agents for Simultaneous Oxidation of NADH and Generation
of Superoxide Radical. Nat. Commun..

[157] Lee C., Park M., Wijesinghe W. C. B., Na S., Lee C. G., Hwang E., Yoon G., Lee J. K., Roh D.-H., Kwon Y. H., Yang J., Hughes S. A., Vince J. E., Seo J. K., Min D., Kwon T.-H. (2024). Oxidative Photocatalysis
on Membranes Triggers Non-Canonical Pyroptosis. Nat. Commun..

[158] Wu Y., Liu Q., Li S., Yu W., Fan H., Yao S., He W., Guo Z., Chen Y. (2024). Mitochondria Targeting
Photoredox Catalyst-Induced Pyroptosis for Enhanced Immunotherapy
against Hypoxic Tumor Cells. Chem. Eng. J..

[159] Li Q., Yan C., Zhang P., Wang P., Wang K., Yang W., Cheng L., Dang D., Cao L. (2024). Tetraphenylethene-Based
Molecular Cage with Coenzyme FAD: Conformationally Isomeric Complexation
toward Photocatalysis-Assisted Photodynamic Therapy. J. Am. Chem. Soc..

[160] Zhou K., Du L., Ding R., Xu L., Shi S., Wang S., Wang Z., Zhang G., He G., Zhao Z., Tang B. Z. (2024). Photocatalytic Therapy via Photoinduced
Redox Imbalance in Biological System. Nat. Commun..

[161] Kim J., Xu Y., Lim J. H., Lee J. Y., Li M., Fox J. M., Vendrell M., Kim J. S. (2025). Bioorthogonal Activation
of Deep Red Photoredox Catalysis Inducing Pyroptosis. J. Am. Chem. Soc..

[162] Yao S., Xu F., Wang Y., Shang J., Li S., Xu X., Liu Z., He W., Guo Z., Chen Y. (2025). Photoinduced
Synergism of Ferroptosis/Pyroptosis/Oncosis by an O_2_-Independent
Photocatalyst for Enhanced Tumor Immunotherapy. J. Am. Chem. Soc..

[163] Li D., Wen G., Wang H., Ren Q., Wang D., Dao A., Huang H., Zhang P. (2025). Photoredox-Mediated Immunotherapy
Utilizing Rhenium­(I) Photocatalysts with Electron Donor–Acceptor–Donor
Configuration. J. Med. Chem..

[164] Pham T. C., Nguyen V.-N., Choi Y., Lee S., Yoon J. (2021). Recent Strategies to Develop Innovative Photosensitizers
for Enhanced
Photodynamic Therapy. Chem. Rev..

[165] Tummers W. S., Warram J. M., van den
Berg N. S., Miller S. E., Swijnenburg R.-J., Vahrmeijer A. L., Rosenthal E. L. (2018). Recommendations for Reporting on
Emerging Optical Imaging
Agents to Promote Clinical Approval. Theranostics.

[166] Dagogo-Jack I., Shaw A. T. (2018). Tumour Heterogeneity
and Resistance
to Cancer Therapies. Nat. Rev. Clin. Oncol..

[167] Nestoros E., Sharma A., Kim E., Kim J. S., Vendrell M. (2025). Smart Molecular Designs and Applications
of Activatable
Organic Photosensitizers. Nat. Rev. Chem..

[168] Kwon N., Weng H., Rajora M. A., Zheng G. (2025). Activatable
Photosensitizers: From Fundamental Principles to Advanced Designs. Angew. Chem., Int. Ed..

[169] Pei Y., Pan Y., Zhang Z., Zhu J., Sun Y., Zhang Q., Zhu D., Li G., Bryce M. R., Wang D., Tang B. Z. (2025). Leveraging Tumor Microenvironment
to Boost Synergistic Photodynamic Therapy, Ferroptosis Anti-Tumor
Efficiency Based on a Functional Iridium­(III) Complex. Adv. Sci..

[170] Huang T., Yu Q., Liu S., Zhang K. Y., Huang W., Zhao Q. (2019). Rational Design
of Phosphorescent
Iridium­(III) Complexes for Selective Glutathione Sensing and Amplified
Photodynamic Therapy. ChemBioChem..

[171] Liu J., Prentice A. W., Clarkson G. J., Woolley J. M., Stavros V. G., Paterson M. J., Sadler P. J. (2023). A Concerted
Redox- and Light-Activated
Agent for Controlled Multimodal Therapy against Hypoxic Cancer Cells. Adv. Mater..

[172] Xu J.-W., Lee L. C.-C., Yip A. M.-H., Xu G.-X., Leung P. K.-K., Lo K. K.-W. (2025). Luminescent
Iridium­(III) 2-Cyanobenzothiazole
Complexes as Site-Specific Labels to Afford Peptide-Based Phosphorogenic
Probes and Hydrogels for Enzyme Activity Sensing, Cancer Imaging and
Photodynamic Therapy. Inorg. Chem. Front..

[173] Ning S., Yao Y., Feng X., Tian Y. (2025). Recent Advances
in Developing Bioorthogonally Activatable Photosensitizers for Photodynamic
Therapy. Eur. J. Med. Chem..

[174] Oliveira B. L., Guo Z., Bernardes G. J. L. (2017). Inverse
Electron Demand Diels–Alder Reactions in Chemical Biology. Chem. Soc. Rev..

[175] Bilodeau D. A., Margison K. D., Serhan M., Pezacki J. P. (2021). Bioorthogonal
Reactions Utilizing Nitrones as Versatile Dipoles in Cycloaddition
Reactions. Chem. Rev..

[176] Choi A. W.-T., Tso K. K.-S., Yim V. M.-W., Liu H.-W., Lo K. K.-W. (2015). Modification of 1,2,4,5-Tetrazine
with Cationic Rhenium­(I)
Polypyridine Units to Afford Phosphorogenic Bioorthogonal Probes with
Enhanced Reaction Kinetics. Chem. Commun..

[177] Lee L. C.-C., Lau J. C.-W., Liu H.-W., Lo K. K.-W. (2016). Conferring
Phosphorogenic Properties on Iridium­(III)-Based Bioorthogonal Probes
through Modification with a Nitrone Unit. Angew.
Chem., Int. Ed..

[178] Tang T. S.-M., Liu H.-W., Lo K. K.-W. (2016). Structural Manipulation
of Ruthenium­(II) Polypyridine Nitrone Complexes to Generate Phosphorogenic
Bioorthogonal Reagents for Selective Cellular Labeling. Chem.—Eur. J..

[179] Li S. P.-Y., Yip A. M.-H., Liu H.-W., Lo K. K.-W. (2016). Installing
an Additional Emission Quenching Pathway in the Design of Iridium­(III)-Based
Phosphorogenic Biomaterials for Bioorthogonal Labelling and Imaging. Biomaterials.

[180] Tang T. S.-M., Liu H.-W., Lo K. K.-W. (2017). Monochromophoric
Iridium­(III) Pyridyl–Tetrazine Complexes as a Unique Design
Strategy for Bioorthogonal Probes with Luminogenic Behavior. Chem. Commun..

[181] Leung P. K.-K., Lo K. K.-W. (2020). Modulation of Emission and Singlet
Oxygen Photosensitisation in Live Cells Utilising Bioorthogonal Phosphorogenic
Probes and Protein Tag Technology. Chem. Commun..

[182] Leung P. K.-K., Lee L. C.-C., Yeung H. H.-Y., Io K.-W., Lo K. K.-W. (2021). Bioorthogonal Control of the Phosphorescence and Singlet
Oxygen Photosensitisation Properties of Iridium­(III) Tetrazine Complexes. Chem. Commun..

[183] Mak E. C.-L., Chen Z., Lee L. C.-C., Leung P. K.-K., Yip A. M.-H., Shum J., Yiu S.-M., Yam V. W.-W., Lo K. K.-W. (2024). Exploiting
the Potential of Iridium­(III) *bis*-Nitrone Complexes
as Phosphorogenic Bifunctional Reagents for Phototheranostics. J. Am. Chem. Soc..

[184] Yip A. M.-H., Lai C. K.-H., Yiu K. S.-M., Lo K. K.-W. (2022). Phosphorogenic
Iridium­(III) *bis*-Tetrazine Complexes for Bioorthogonal
Peptide Stapling, Bioimaging, Photocytotoxic Applications, and the
Construction of Nanosized Hydrogels. Angew.
Chem., Int. Ed..

[185] Shum J., Lee L. C.-C., Chiang M. W.-L., Lam Y.-W., Lo K. K.-W. (2023). A Concerted Enzymatic and Bioorthogonal Approach for
Extracellular and Intracellular Activation of Environment-Sensitive
Ruthenium­(II)-Based Imaging Probes and Photosensitizers. Angew. Chem., Int. Ed..

[186] Xu G.-X., Lee L. C.-C., Leung P. K.-K., Mak E. C.-L., Shum J., Zhang K. Y., Zhao Q., Lo K. K.-W. (2023). Bioorthogonal
Dissociative Rhenium­(I) Photosensitisers for Controlled Immunogenic
Cell Death Induction. Chem. Sci..

[187] Yang M., Huang J., Fan J., Du J., Pu K., Peng X. (2020). Chemiluminescence for Bioimaging
and Therapeutics:
Recent Advances and Challenges. Chem. Soc. Rev..

[188] Blau R., Shelef O., Shabat D., Satchi-Fainaro R. (2023). Chemiluminescent
Probes in Cancer Biology. Nat. Rev. Bioeng..

[189] Zhu J.-H., Gu M., Chen Y., Li M., Chen X., Yoon J., Peng X. (2025). Chemiluminescent Transition
Metal Complexes: Mechanisms and Applications. Coord. Chem. Rev..

[190] Hercules D. M., Lytle F. E. (1966). Chemiluminescence from Reduction
Reactions. J. Am. Chem. Soc..

[191] Büchel G. E., Carney B., Shaffer T. M., Tang J., Austin C., Arora M., Zeglis B. M., Grimm J., Eppinger J., Reiner T. (2016). Near-Infrared Intraoperative
Chemiluminescence
Imaging. ChemMedChem..

[192] Zhu J.-H., He X., Wu Y., Huang H., Yang D., Li J., Gu M., Wang L., Li M., Chen X., Peng X. (2025). Cyclometalated
Iridium­(III) Schiff
Base Complexes for Chemiluminogenic Bioprobes. Angew. Chem., Int. Ed..

[193] Wang Z., Zhu B., Nie W., Zhang L., Xiao N., Zhang Q., Wu Z., Shi C., Zhu W., Liu Q., Zhu D., Bryce M. R., Ren L., Tang B. Z. (2025). Endogenous Near-Infrared Chemiluminescence: Imaging-Guided
Non-Invasive Thrombolysis and Anti-Inflammation Based on a Heteronuclear
Transition Metal Complex. Adv. Sci..

[194] Kagalwala H. N., Gerberich J., Smith C. J., Mason R. P., Lippert A. R. (2022). Chemiluminescent
1,2-Dioxetane Iridium Complexes for
Near-Infrared Oxygen Sensing. Angew. Chem.,
Int. Ed..

[195] Cui X., Li X., Peng C., Qiu Y., Shi Y., Liu Y., Fei J.-F. (2023). Beyond External Light: On-Spot Light Generation or
Light Delivery for Highly Penetrated Photodynamic Therapy. ACS Nano.

[196] Da X., Xu Y., Wang L., Liu X., Peng Y., Wu Y., Zhou W., Wang W., Wang X., Zhou Q. (2024). A Self-Enhanced
Chemiexcited PDT System for Targeted and Efficient Treatment of Deeply
Seated Tumors. Inorg. Chem. Front..

[197] Liu S., Chen H., Wu Q., Sun Y., Pei Y., Wang Z., Zhu D., Li G., Bryce M. R., Chang Y. (2024). Self-Chemiluminescence-Triggered
Ir­(III) Complex Photosensitizer
for Photodynamic Therapy against Hypoxic Tumor. Inorg. Chem..

[198] Green O., Eilon T., Hananya N., Gutkin S., Bauer C. R., Shabat D. (2017). Opening a Gateway for Chemiluminescence
Cell Imaging: Distinctive Methodology for Design of Bright Chemiluminescent
Dioxetane Probes. ACS Cent. Sci..

[199] Tannous R., Shelef O., Gutkin S., Davaid M., Leirikh T., Ge L., Jaber Q., Zhou Q., Ma P., Fridman M., Spitz U., Houk K. N., Shabat D. (2024). Spirostrain-Accelerated
Chemiexcitation of Dioxetanes Yields Unprecedented Detection Sensitivity
in Chemiluminescence Bioassays. ACS Cent. Sci..

[200] Kagalwala H. N., Reeves R. T., Lippert A. R. (2022). Chemiluminescent
Spiroadamantane-1,2-Dioxetanes: Recent Advances in Molecular Imaging
and Biomarker Detection. Curr. Opin. Chem. Biol..

